# Functionalized Oxoindolin Hydrazine Carbothioamide Derivatives as Highly Potent Inhibitors of Nucleoside Triphosphate Diphosphohydrolases

**DOI:** 10.3389/fphar.2020.585876

**Published:** 2020-11-30

**Authors:** Saira Afzal, Mariya al-Rashida, Abdul Hameed, Julie Pelletier, Jean Sévigny, Jamshed Iqbal

**Affiliations:** ^1^ Centre for Advanced Drug Research, COMSATS University Islamabad, Abbottabad Campus, Abbottabad, Pakistan; ^2^ Department of Chemistry, Forman Christian College (A Chartered University), Lahore, Pakistan; ^3^ Centre de Recherche du CHU de Québec–Université Laval, Québec City, QC, Canada; ^4^ Département de Microbiologie-Infectiologie et d’Immunologie, Faculté de Médecine, Université Laval, Québec City, QC, Canada

**Keywords:** carbothioamide, ectonucleotidases, nucleoside triphosphate diphosphohydrolase-3, oxoindolin hydrazine, molecular docking

## Abstract

Ectonucleoside triphosphate diphosphohydrolases (NTPDases) are ectoenzymes that play an important role in the hydrolysis of nucleoside triphosphate and diphosphate to nucleoside monophosphate. NTPDase1, -2, -3 and -8 are the membrane bound members of this enzyme family that are responsible for regulating the levels of nucleotides in extracellular environment. However, the pathophysiological functions of these enzymes are not fully understood due to lack of potent and selective NTPDase inhibitors. Herein, a series of oxoindolin hydrazine carbothioamide derivatives is synthesized and screened for NTPDase inhibitory activity. Four compounds were identified as selective inhibitors of *h*-NTPDase1 having IC_50_ values in lower micromolar range, these include compounds **8b** (IC_50_ = 0.29 ± 0.02 µM), **8e** (IC_50_ = 0.15 ± 0.009 µM), **8f** (IC_50_ = 0.24 ± 0.01 µM) and **8l** (IC_50_ = 0.30 ± 0.03 µM). Similarly, compound **8k** (IC_50_ = 0.16 ± 0.01 µM) was found to be a selective *h*-NTPDase2 inhibitor. In case of *h*-NTPDase3, most potent inhibitors were compounds **8c** (IC_50_ = 0.19 ± 0.02 µM) and **8m** (IC_50_ = 0.38 ± 0.03 µM). Since NTPDase3 has been reported to be associated with the regulation of insulin secretion, we evaluated our synthesized NTPDase3 inhibitors for their ability to stimulate insulin secretion in isolated mice islets. Promising results were obtained showing that compound **8m** potently stimulated insulin secretion without affecting the NTPDase3 gene expression. Molecular docking studies of the most potent compounds were also carried out to rationalize binding site interactions. Hence, these compounds are useful tools to study the role of NTPDase3 in insulin secretion.

## Introduction

Glucose acts as a natural insulin secretagogue and maintains the blood glucose level. In response to an increase in blood glucose level, pancreatic beta cells release insulin to maintain glucose levels (Chen et al., 2018; Stuhlmann et al., 2018). However, this insulin secretion is not an isolated event, and is followed by release of other important components like peptides, Zn^+2^, adenosine triphosphate (ATP) and other related nucleotides (Tozzi et al., 2018; Vakilian et al., 2019). Among them, ATP is an important signaling molecule that amplifies the glucose stimulated insulin secretion by activating purinergic (P2X and P2Y) receptors, present on the surface of β-cell membrane (Cieślak and Cieślak, 2017). P2X receptors are ionotropic receptors that are subdivided into seven types (P2X1 to P2X7). They have been associated with the transmembrane flow of cations such as Na^+^, Ca^+2^, and K^+^ ions (Kasuya et al., 2017a; Kasuya et al., 2017b; Giuliani et al., 2019). On the other hand, P2Y receptors are metabotropic receptors that are subdivided into eight types (P2Y_1_, P2Y_2_, P2Y_4_, P2Y_6_, P2Y_11_, P2Y_12_, P2Y_13_, and P2Y_14_) and have been linked to Ca^+2^ mobilization and generation or inhibition of cyclic adenosine monophosphate (cAMP) (Dou et al., 2018; Lu et al., 2019). In pancreatic β-cells, both receptor types have been reported to be involved in the regulation of insulin secretion; however, their mode of action is slightly different (Galicia-Garcia et al., 2020). In this regard, P2X receptor activation by ATP leads to Ca^+2^ flux that is believed to be responsible for P2X-mediated insulin secretion (Solini and Novak, 2019). Whereas, activation of P2Y receptors stimulates the inositol triphosphate, which in turn results in transient elevation of intracellular concentration of Ca^+2^ (Bartley et al., 2019).

The extracellular levels of ATP and related nucleotides are controlled by a cascade of enzymes known as ectonucleotidases. This enzymatic cascade is composed of four main families, including NTPDases (ectonucleoside triphosphate diphosphohydrolases), APs or ALPs (alkaline phosphatases), NPPs (nucleotide pyrophosphatases/phosphodiesterases) and e-5′-NT (ecto-5′-nucleotidase) (Lee et al., 2017; Lee and Müller, 2017; Tozzi and Novak, 2017; Grković et al., 2019; Nabinger et al., 2020). These are plasma membrane bound ecto-enzymes and regulate the availability of ligands (nucleotides and hydrolysis products thereof) for P1 and P2 receptors. Among them, NTPDase is an important member contributing to the hydrolysis of nucleoside tri- and diphosphates (Longhi et al., 2017; Bagatini et al., 2018). The NTPDase family comprises of eight members or isozymes (NTPDase1-8), these isozymes differ in their catalytic properties and show varying substrate preference (Allard et al., 2017; Zhong et al., 2017). NTPDase1, -2, -3, and -8 are membrane bound isoforms that play an important role in purinergic signaling (Castilhos et al., 2018; Bertoni et al., 2020 ) whereas NTPDase4, -5, -6, and -7 are expressed within intracellular organelles (Zhong et al., 2017; Paes‐Vieira et al., 2018).

NTPDase3 is abundantly present in the pancreatic β-cells where it has been reported to play an important role in the regulation of insulin secretion (Bartley et al., 2019; Saunders et al., 2019). In this context, a study was conducted where 1-naphthol-3, 6-disulfonic acid (BG0136) and 8,8′-[Carbonylbis(imino-4,1-phenylenecarbonylimino-4,1-phenylenecarbonylimino)]bis-1,3,5-naphthalenetrisulfonic acid (NF279) were used to explore the role of NTPDase3 in insulin secretion (Lavoie et al., 2010). Similarly, in another study 6-*N,N*-Diethyl-d-*β*-*γ*-dibromomethylene adenosine triphosphate (ARL67156) was used for this purpose and it was found that NTPDase3 was involved in the modulation of insulin release (Syed et al., 2013). These data highlight the potential (important) role of NTPDase3 in insulin secretion and necessitate the need for further detailed investigations to support these claims. However, to the best of our knowledge, there is still a lack of studies focusing on this aspect of NTPDase3. Keeping in view this scarcity of data, we synthesized a class of oxoindolin hydrazine carbothioamide derivatives as NTPDase inhibitors. The NTPDase3 inhibitors were further analyzed for their effects on insulin secretion in mice pancreatic islets.

## Materials and Methods

### Chemical Synthesis

#### General Comments

All reagents were obtained from commercial sources and used without further purification, unless stated otherwise. Reaction progress was monitored by thin layer chromatography, using aluminum sheets precoated with silica gel 60 F_254_ (200 μm, Merck, Darmstadt, Germany). Melting points were determined on Weiss Gallenkamp melting point apparatus (Loughborough, England) and are uncorrected. IR spectra were recorded on Perkin Elmer BX-II spectrometer (Waltham, United States). ^1^H and ^13^C spectra were obtained using Bruker AM-300 spectrophotometer (Billerica, United States). Chemical shifts were described in parts per million. Mass spectra were recorded on an Applied Biosystems API 2000 mass spectrometer (Darmstadt, Germany).

#### General Procedure for the Synthesis of Substituted Phenylhydrazine Carbothioamides (7a-m)

Excess of hydrazine hydrate was dissolved in ethanol and a solution of appropriate isothiocyanate (1 mMol) was prepared in ethanol. The isothiocyanate solution was added drop wise to hydrazine solution. The reaction was conducted on ice bath and stirred for half an hour. During the course of reaction, precipitates were formed which were filtered, washed and then dried.

#### General Procedure for the Synthesis of Oxoindolin-Ylidene Phenylhydrazine Carbothioamides (8a-m)

A solution of isatin (1 mMol, 0.147 g) was prepared in ethanol and few drops of acetic acid were added. Resulting solution was refluxed for 4 to 5 h with equimolar amount of an appropriate intermediate (**7a-m**), synthesized during last step. Progress of reaction was monitored by thin layer chromatography. At the end of reaction, the precipitated solid was filtered and washed with ethanol (Pervez et al., 2009).

(*Z*)-2-(2-oxoindolin-3-ylidene)-*N*-phenylhydrazine-1-carbothioamide (8a)

Obtained as yellow solid, Yield = 78%, mp = 245–247°C; IR (KBR): 3,280–3,172 (NH stretching), 1,691 (C = O), 1,603 (C = N), 1,560 (NH bending), 1,161 (C = S), cm^−1^; ^**1**^ **HNMR** (300 MHz, DMSO-*d6*): δ (ppm) 12.80 (s, 1H, N-H), 11.26 (s, 1H, N-H), 10.82 (s, 1H, N-H), 7.78 (d, *J* = 7.2, 1H, Ar-H), 7.61 (d, *J* = 7.8, 2H, Ar-H), 7.39 (m, 3H, Ar-H), 7.27 (t, *J* = 7.35, 1H, Ar-H) 7.11 (t, *J* = 7.5, 1H, Ar-H), 6.95 (d, *J* = 7.8, 1H, Ar-H); ^**13**^ **C-NMR** (75 MHz, DMSO*-d6*) δ (ppm): 175.60, 163.10, 146.23, 135.16, 132.88, 131.10, 128.06, 126.23, 125.45, 123.12, 122.18, 120.05, 111.67; **LC-MS** (*m/z*): positive mode 297 [M + H]^+^. Purity determined by HPLC-UV (254 nm)-ESI-MS: 97.26%

(*Z*)-*N*-(4-nitrophenyl)-2-(2-oxoindolin-3-ylidene)hydrazine-1-carbothioamide (8b)

Obtained as yellow solid, Yield = 86%, mp = 271–272°C; IR (KBR): 3,315–3,222 (NH stretching), 1,694 (C = O), 1,600 (C = N), 1,556 (NH bending), 1,168 (C = S), cm^−1^; ^**1**^ **HNMR** (300 MHz, DMSO-*d6*): δ (ppm) 13.01 (s, 1H, N-H), 11.31 (s, 1H, N-H), 11.12 (s, 1H, N-H), 8.29 (m, 2H, Ar-H), 8.09 (m, 2H, Ar-H), 7.78 (d, *J* = 7.2, 1H, Ar-H), 7.40 (td, *J* = 7.8, 1.2 Hz, 1H, Ar-H), 7.13 (t, *J* = 7.5, 1H, Ar-H), 6.96 (d, *J* = 7.8, 1H, Ar-H); ^**13**^ **C-NMR** (75 MHz, DMSO*-d6*) δ (ppm): 176.53, 163.20, 145.12, 144.60, 143.24, 133.79, 132.30, 125.24, 124.47, 122.93, 122.07, 120.15, 111.69; **LC-MS** (*m/z*): positive mode 342 [M + H]^+^. Purity determined by HPLC-UV (254 nm)-ESI-MS: 97.18%

(*Z*)-*N*-(4-chlorophenyl)-2-(2-oxoindolin-3-ylidene)hydrazine-1-carbothioamide (8c)

Obtained as yellow solid, Yield = 85% mp = 240–244°C; IR (KBR): 3,315–3,210 (NH stretching), 1,692 (C = O), 1,591 (C = N), 1,527 (NH bending), 1,164 (C = S), cm^−1^; ^**1**^ **HNMR** (300 MHz, DMSO-*d6*): δ (ppm) 12.84 (s, 1H, N-H), 11.27 (s, 1H, N-H), 10.86 (s, 1H, N-H), 7.76 (d, *J* = 7.2, 1H, Ar-H), 7.66 (m, 2H, Ar-H), 7.49 (m, 2H, Ar-H), 7.37 (td, *J* = 7.65, 1.1, 1H, Ar-H), 7.11 (td, *J* = 7.5, 0.7, 1H, Ar-H), 6.94 (d, *J* = 7.8, 1H, Ar-H); ^**13**^ **C-NMR** (75 MHz, DMSO*-d6*) δ (ppm): 176.86, 163.17, 143.04, 137.92, 133.06, 132.01, 130.56, 128.79, 127.79, 122.86, 121.88, 120.30, 111.60; **LC-MS** (*m/z*): positive mode 331 [M + H]^+^. Purity determined by HPLC-UV (254 nm)-ESI-MS: 95.63%

(*Z*)-*N*-(4-fluorophenyl)-2-(2-oxoindolin-3-ylidene)hydrazine-1-carbothioamide (8d)

Obtained as yellow solid, Yield = 87%, mp = 255–256°C; IR (KBR): 3,290–3,185 (NH stretching), 1,694 (C = O), 1,610 (C = N), 1,560 (NH bending), 1,166 (C = S), cm^−1^; ^**1**^ **HNMR** (300 MHz, DMSO-*d6*): δ (ppm) 12.81 (s, 1H, N-H), 11.27 (s, 1H, N-H), 10.83 (s, 1H, N-H), 7.75 (d, *J* = 7.5, 1H, Ar-H), 7.60 (dd, *J* = 8.7, 5.1, 2H, Ar-H), 7.37 (t, *J* = 7.35, 1H, Ar-H), 7.26 (t, *J* = 8.85, 2H, Ar-H), 7.11 (t, *J* = 7.5, 1H, Ar-H), 6.94 (d, *J* = 7.8, 1H, Ar-H); ^**13**^ **C-NMR** (75 MHz, DMSO*-d6*) δ (ppm): 177.20, 162.10, 158.88, 142.99, 135.26, 132.89, 131.95, 128.52, 122.86, 121.82, 120.35, 115.74, 111.58; **LC-MS** (*m/z*): positive mode 315 [M + H]^+^. Purity determined by HPLC-UV (254 nm)-ESI-MS: 98.93%

(*Z*)-*N*-(3-methoxyphenyl)-2-(2-oxoindolin-3-ylidene)hydrazine-1-carbothioamide (8e)

Obtained as yellow solid, Yield = 80%, mp = 220–224°C; IR (KBR): 3,240–3,189 (NH stretching), 1,690 (C = O), 1,595 (C = N), 1,526 (NH bending), 1,161 (C = S), cm^−1^; ^**1**^ **HNMR** (300 MHz, DMSO-*d6*): δ (ppm) 12.80 (s, 1H, N-H), 11.27 (s, 1H, N-H), 10.77 (s, 1H, N-H), 7.79 (d, *J* = 7.5, 1H, Ar-H), 7.35 (m, 3H, Ar-H), 7.24 (d, *J* = 7.2, 1H, Ar-H), 7.11 (t, *J* = 7.5, 2H, Ar-H), 6.94 (d, *J* = 7.8, 1H, Ar-H), 6.85 (dd, *J* = 8.1, 2.4, 1H, Ar-H); ^**13**^ **C-NMR** (75 MHz, DMSO*-d6*) δ (ppm): 176.49, 163.17, 159.64, 142.97, 139.99, 132.77, 131.92, 129.59, 127.55, 122.83, 121.92, 120.35, 118.0, 112.0, 111.55, 55.69; **LC-MS** (*m/z*): positive mode 327 [M + H]^+^. Purity determined by HPLC-UV (254 nm)-ESI-MS: 99.02%

(*Z*)-*N*-benzyl-2-(2-oxoindolin-3-ylidene)hydrazine-1-carbothioamide (8f)

Obtained as yellow solid, Yield = 84%, mp = 206–208°C; IR (KBR): 3,271–3,142 (NH stretching), 1,694 (C = O), 1,594 (C = N), 1,535 (NH bending), 1,170 (C = S), cm^−1^; ^**1**^ **HNMR** (300 MHz, DMSO-*d6*): δ (ppm) 12.66 (s, 1H, N-H), 11.22 (s, 1H, N-H), 9.82 (s,1H, N-H), 7.65 (d, *J* = 7.5, 1H, Ar-H), 7.35 (m, 5H, Ar-H), 7.26 (m, 1H, Ar-H), 7.08 (d, *J* = 7.5, 1H, Ar-H), 6.93 (d, *J* = 7.8, 1H, Ar-H), 4.88 (d, *J* = 6, 2H, Alkyl H); ^**13**^ **C-NMR** (75 MHz, DMSO-*d6*) δ (ppm): 178.17, 163.11, 142.82, 138.87, 132.53, 131.72, 128.75, 127.81, 127.48, 122.77, 121.37, 120.42, 111.55, 47.67; **LC-MS** (*m/z*): positive mode 311 [M + H]^+^. Purity determined by HPLC-UV (254 nm)-ESI-MS: 97.81%

(*Z*)-*N*-(3-chlorophenyl)-2-(2-oxoindolin-3-ylidene)hydrazine-1-carbothioamide (8g)

Obtained as yellow solid, Yield = 74%, mp = 235–236°C; IR (KBR): 3,340–3,192 (NH stretching), 1,694 (C=O), 1,594 (C=N), 1,535 (NH bending), 1,170 (C=S), cm^−1^; ^**1**^ **HNMR** (300 MHz, DMSO-*d6*): δ (ppm) 12.86 (s, 1H, N-H), 11.28 (s, 1H, N-H), 10.87 (s, 1H, N-H), 7.79 (t, *J* = 2.1, 1H, Ar-H), 7.76 (s, 1H, Ar-H), 7.65 (dq, *J* = 8.1, 1.2, 1H, Ar-H), 7.45 (t, *J* = 7.95, 1H, Ar-H), 7.35 (m, 2H, Ar-H), 7.12 (t, *J* = 7.35, 1H, Ar-H), 6.95 (d, *J* = 7.8, 1H, Ar-H); ^**13**^ **C-NMR** (75 MHz, DMSO*-d6*) δ (ppm); 176.77, 163.18, 143.09, 140.40, 133.19, 132.91, 132.08, 130.44, 126.28, 125.53, 124.52, 122.87, 121.93, 120.28, 111.62; **LC-MS** (*m/z*): positive mode 331 [M + H]^+^. Purity determined by HPLC-UV (254 nm)-ESI-MS: 98.38%

(*Z*)-2-(2-oxoindolin-3-ylidene)-*N*-(*p*-tolyl)hydrazine-1-carbothioamide (8h)

Obtained as yellow solid, Yield = 79%, mp = 240–242°C; IR (KBR): 3,260–3,130 (NH stretching), 1,698 (C = O), 1,608 (C = N), 1,530 (NH bending), 1,158 (C = S), cm^−1^; ^**1**^ **HNMR** (300 MHz, DMSO-*d6*): δ (ppm) 12.77 (s, 1H, N-H), 11.25 (s, 1H, N-H), 10.75 (s, 1H, N-H), 7.77 (d, *J* = 7.2, 1H, Ar-H), 7.47 (d, *J* = 8.1, 2H, Ar-H), 7.37 (td, *J* = 7.8, 1.2, 1H, Ar-H), 7.22 (d, *J* = 8.4, 2H, Ar-H), 7.10 (dt, *J* = 7.65, 0.7, 1H, Ar-H), 6.94 (d, *J* = 7.8, 1H, Ar-H), 2.47 (s, 2H, Alkyl H); ^**13**^ **C-NMR** (75 MHz, DMSO-*d6*) δ (ppm); 176.79, 163.17, 142.92, 136.36, 135.83, 132.61, 131.85, 129.31, 126.04, 122.82, 121.84, 120.41, 111.54, 21.0.

(*Z*)-*N*-(2,6-dimethylphenyl)-2-(2-oxoindolin-3-ylidene)hydrazine-1-carbothioamide (8i)

Obtained as yellow solid, Yield = 83%, mp = 250-252°C; IR (KBR): 3,250–3,155 (NH stretching), 1,688 (C = O), 1,619 (C = N), 1,520 (NH bending), 1,174 (C = S), cm^−1^; ^**1**^ **HNMR** (300 MHz, DMSO-*d6*): δ (ppm) 12.76 (s, 1H, N-H), 11.23 (s, 1H, N-H), 10.61 (s, 1H, N-H), 7.73 (d, *J* = 7.5, 1H, Ar-H), 7.36 (t, *J* = 7.65, 1H, Ar-H), 7.17 (m, 3H, Ar-H), 7.09 (t, *J* = 7.5, 1H, Ar-H), 6.94 (d, *J* = 7.8, 1H, Ar-H), 2.20 (s, 6H, Alkyl H); ^**13**^ **C-NMR** (75 MHz, DMSO-*d6*) δ (ppm); 177.43, 163.12, 142.92, 136.82, 136.52, 132.58, 131.77, 128.31, 127.89, 122.81, 121.71, 120.49, 111.51, 18.32; **LC-MS** (*m/z*): positive mode 325 [M + H]^+^. Purity determined by HPLC-UV (254 nm)-ESI-MS: 98.27%

(*Z*)-*N*-(2,5-dimethoxyphenyl)-2-(2-oxoindolin-3-ylidene)hydrazine-1-carbothioamide (8j)

Obtained as yellow solid, Yield = 74%, mp = 245–248°C; IR (KBR): 3,285–3,170 (NH stretching), 1,690 (C = O), 1,606 (C = N), 1,545 (NH bending), 1,164 (C = S), cm^−1^; ^**1**^ **HNMR** (300 MHz, DMSO-*d6*): δ (ppm) 12.81 (s, 1H, N-H), 11.27 (s, 1H, N-H), 10.43 (s, 1H, N-H), 7.77 (d, *J* = 2.7, 1H, Ar-H), 7.65 (d, *J* = 7.2, 1H, Ar-H), 7.38 (td, *J* = 7.65, 1.1, 1H, Ar-H), 7.09 (m, 2H, Ar-H), 6.95 (d, *J* = 7.8, 1H, Ar-H), 6.82 (dd, *J* = 9.0, 3.0, 1H, Ar-H), 3.84 (s, 3H, Alkyl H), 3.72 (s, 3H, Ar-H); ^**13**^ **C-NMR** (75 MHz, DMSO-*d6*) δ (ppm): 175.64, 163.17, 153.06, 146.68, 143.09, 132.87, 132.05, 128.06, 123.00, 121.41, 120.15, 112.78, 111.64, 111.53, 56.84, 55.95; **LC-MS** (*m/z*): positive mode 357 [M + H]^+^. Purity determined by HPLC-UV (254 nm)-ESI-MS: 95.39%

(*Z*)-*N*-(4-methoxyphenyl)-2-(2-oxoindolin-3-ylidene)hydrazine-1-carbothioamide (8k)

Obtained as yellow solid, Yield = 80%, mp = 255–260°C; IR (KBR): 3,270–3,160 (NH stretching), 1,692 (C = O), 1,623 (C = N), 1,546 (NH bending), 1,162 (C = S), cm^−1^; ^**1**^ **HNMR** (300 MHz, DMSO-*d6*): δ (ppm) 12.75 (s, 1H, N-H), 11.25 (s, 1H, N-H), 10.73 (s, 1H, N-H), 7.76 (d, *J* = 7.5, 1H, Ar-H), 7.46 (d, *J* = 8.4, 2H, Ar-H), 7.37 (td, *J* = 7.8, 1.0, 1H, Ar-H), 7.10 (t, *J* = 7.2, 1H, Ar-H), 6.97 (t, *J* = 9.75, 3H, Ar-H), 3.78 (s, 3H, Alkyl-H); ^**13**^ **C-NMR** (75 MHz, DMSO*-d6*) δ (ppm): 177.0, 163.16, 157.85, 142.89, 132.52, 131.80, 131.74, 127.73, 122.82, 121.79, 120.44, 114.03, 111.54, 55.75; **LC-MS** (*m/z*): positive mode 327 [M + H]^+^. Purity determined by HPLC-UV (254 nm)-ESI-MS: 98.73%

(*Z*)-*N*-(2,6-difluorophenyl)-2-(2-oxoindolin-3-ylidene)hydrazine-1-carbothioamide (8l)

Obtained as yellow solid, Yield = 75%, mp = 243–244°C; IR (KBR, cm^−1^): 3,264–3,170 (NH stretching), 1,692 (C = O), 1,598 (C = N), 1,555 (NH bending), 1,170 (C = S), cm^−1^; ^**1**^ **HNMR** (300 MHz, DMSO-*d6*) δ (ppm): 12.96 (s, 1H, N-H), 11.28 (s, 1H, N-H), 10.57 (s, 1H, N-H), 7.69 (d, *J* = 7.5, 1H, Ar-H), 7.49 (m, 1H, Ar-H), 7.39 (td, *J* = 7.65, 1.1, 1H, Ar-H), 7.26 (t, *J* = 9.9, 2H, Ar-H), 7.12 (t, *J* = 7.65, 1H, Ar-H), 6.95 (d, *J* = 7.5, 1H, Ar-H); ^**13**^ **C-NMR** (75 MHz, DMSO-*d6*) δ (ppm): 179.09, 163.11, 157.42, 143.27, 133.83, 132.18, 130.15, 122.94, 121.72, 120.26, 116.56, 112.49, 111.67; **LC-MS** (*m/z*): positive mode 333 [M + H]^+^. Purity determined by HPLC-UV (254 nm)-ESI-MS: 97%

(*Z*)-*N*-ethyl-2-(2-oxoindolin-3-ylidene)hydrazine-1-carbothioamide (8m)

Obtained as yellow solid, Yield = 84%, mp = 190–194°C; IR (KBR): 3,315–3,281 (NH stretching), 1,686 (C = O), 1,619 (C = N), 1,541 (NH bending), 1,161 (C = S), cm^−1^; ^**1**^ **HNMR** (300 MHz, DMSO-*d6*) δ (ppm): 12.96 (s, 1H, N-H), 11.28 (s, 1H, N-H), 10.57 (s, 1H, N-H), 7.65 (d, *J* = 7.2, 1H, Ar-H), 7.35 (td, *J =* 7.72, 1.1, 1H, Ar-H), 7.10 (td, *J* = 7.57, 0.7, 1H, Ar-H), 6.93 (d, *J* = 7.8, 1H, Ar-H), 3.64 (m, 2H, CH_2_), 1.19 (t, *J* = 7.05, 3H, CH_3_); ^**13**^ **C-NMR** (75 MHz, DMSO*-d6*) δ (ppm): 177.08, 163.09, 142.71, 132.11, 131.61, 122.77, 121.27, 120.44, 111.52, 39.51, 14.52; **LC-MS** (*m/z*): positive mode 249 [M + H]^+^. Purity determined by HPLC-UV (254 nm)-ESI-MS: 98.56%

### Biological Protocols

#### Cell Transfection and Protein Preparation

COS-7 cells were transfected with plasmids expressing human or mouse NTPDases1, -2, -3, and -8 as previously described (Levesque et al., 2007; Lecka et al., 2014) Briefly, the confluent cells were transferred to Dulbecco’s Modified Eagle’s Medium containing no fetal bovine serum. Then, these cells were incubated with plasmid DNA (6 µg) and Lipofectamine reagent (24 µl) at 37°C. After 5 h, transfection was terminated with the addition of an equal volume of DMEM/F-12 (supplemented with 20% FBS) and cells were collected by harvesting after 40–72 h.

To prepare protein extracts, transfected COS-7 cells were washed three times with tris-saline buffer while temperature was maintained at 4°C. Then cells were scraped and resuspended in harvesting buffer containing 95 mM NaCl, 45 mM tris and 0.1 mM phenylmethylsulfonyl fluoride (pH 7.5). Cells were again washed twice by centrifugation for 5 min at 300 × g at 4°C and then resuspended in harvesting buffer (supplemented with 10 μg ml aprotinin). Following sonication, cellular debris was isolated by 10 min centrifugation at 300 g at 4°C. Subsequently, supernatant was removed very carefully and protein concentration was determined by using Bradford microplate assay while using the bovine serum albumin as a standard (Bradford, 1976).

#### Nnucleoside Ttriphosphate Ddiphosphohydrolases Activity Assay

The inhibitory effects of synthesized compounds on *h*-NTPDase1, -2, -3, and -8 were determined as described previously, although with slight modifications (Martín-Satué et al., 2009). Assay was conducted in a reaction medium containing 50 mM tris-HCl and 5 mM CaCl_2_ (pH 7.4). All the test compounds were dissolved in dimethyl sulfoxide (DMSO, 10%) and initially screened at a concentration of 100 µM. A reaction mixture containing 55 µl of assay buffer, 10 µl of test compound solution and 10 µl of *h*-NTPDase1 (58 ng/well) or *h*-NTPDase2 (79 ng/well) or *h*-NTPDase3 (163 ng/well) or *h*-NTPDase8 (66 ng/well) was incubated at 37°C for 10 min. Following the incubation, a 10 µl solution of adenosine triphosphate (ATP, 100 µm) was added as substrate and reaction mixture was again incubated for 15 min at 37°C. The reaction was stopped by adding malachite green reagent (15 µl) and placed at room temperature for 4 to 6 min. Finally, absorbance was measured at 630 nm by using Omega FLUOstar microplate reader (BMG Labtech, Ortenberg, Germany) and % inhibitions were calculated. For compounds showing more than 50% inhibition of any isozyme, dose response curves were generated and their IC_50_ values were determined. Three separate enzyme inhibition assays were carried out in triplicate and dose-response curve were generated by using PRISM 5.0 (GraphPad, San Diego, United States).

Same procedure was adopted to determine the inhibitory effects of compounds on mouse NTPDases (*m*-NTPDases). However, the amount of protein used was as follows: *m*-NTPDase1 (82 ng/well), *m*-NTPDase2 (57 ng/well), *m*-NTPDase3 (110 ng/well) and *m*-NTPDase8 (79 ng/well)

#### Islets Isolation

Six to eight week old BALB/c mice, weighing 30–40 g, were used in the study. These mice were obtained from the animal house of COMSATS University Islamabad, Abbottabad Campus and placed under standard temperature (25 ± 2°C) and humidity (50–55%) conditions with an alternate 12-h light/dark cycle. All the experimental animals were provided with a continuous supply of food and water. The animals were handled according to internationally accepted guidelines of animal care and use. Moreover, this study was reviewed and approved by Research Ethics Committee, Department of Pharmacy, COMSATS University Islamabad, Abbottabad Campus. (Protocol number: PHM.Eth./CS-M01-020-1609)

The islets were isolated from mice pancreas as previously described, by a process involving collagenase digestion (Hameed et al., 2018). Briefly, mice were anesthetized with sodium thiopental (30 mg kg) and sacrificed by cervical dislocation to block the flow of blood. Immediately, mice were transferred to biological hood and their abdominal cavity was opened completely by making an incision on abdomen. In order to stop the bile flow toward duodenum, ampulla was located and clamped under dissection microscope. Then, distention of pancreas was carried out by injecting 3 ml of collagenase solution (1 mg/ml) through common bile duct. The distended pancreas was removed as a whole, transferred to a 50 ml tube and digested in collagenase solution at 37°C. After 15 min, digestion was stopped by placing the tube on ice and 20 ml of Hank’s Balanced Salt Solution (HBSS) was added to it. Thus the digested islets were washed with HBSS (two to three times) by centrifugation (1,000 rpm) for 1 min at 4°C. Finally, islets were purified by passing through a cell strainer (70 µm) and isolated manually (by hand picking) under stereomicroscope (Euromex, Arnhem, Netherlands). All the purification and isolation procedures were carried out in HBSS, containing no magnesium, calcium and phenol red.

#### Measurement of Insulin Secretion

Krebs-Ringer bicarbonate buffer (KRBB) was used as an incubation medium for islets and was composed of NaCl (118 mM), KCl (4.7 mM), CaCl_2_ (1.9 mM), MgSO_4_ (1.2 mM), NaHCO_3_ (25 mM), 4-(2-hydroxyethyl)-1-piperazineethanesulfonic acid (HEPES, 10 mM) and bovine serum albumin (0.1%). The pH of KRBB was maintained at 7.4. The isolated islets were size-matched and pre-incubated (3 islets/tube) for 45 min at 37°C in KRBB, supplemented with 3 mM glucose. Thereafter, incubation medium was replaced with fresh KRBB (containing 16.7 mM glucose) and islets were incubated for 60 min at 37°C with or without test substance(s). After incubation, supernatant was collected and stored at −40°C until further use. Finally, collected samples were properly diluted and insulin secretion was estimated with ultra-sensitive mouse insulin ELISA kit (Crystal Chem Inc., Downers Grove, United States) according to the manufacturer’s instructions. The absorbance was measured at 450 nm and insulin secretion was normalized for number of islets used. (Hameed et al., 2018).

#### Ectonucleotidase Activity of Islets

##### Preparation of Islet Homogenate

Islets were isolated and homogenized, according to the previously reported method (Hedeskov and Capito, 1974). After isolation, islets were washed thrice with HBSS and suspended in an ice-cold buffer containing sucrose (0.25 M), ethylenediaminetetraacetic acid (EDTA,1 mM) and tris-HCl (5 mM), maintained at pH 7.0. The resulting suspension was diluted with assay buffer, composed of tris-HCl (50 mM) and CaCl_2_ (5 mM). Afterward, these islets were sonicated for 30 s leading to disruption of islets and formation of a homogenate. Cellular debris was removed by centrifugation at 15,000 rpm for 10 min (4°C). The supernatant was collected and stored on ice. Protein concentration was estimated by Bradford method, using bovine serum albumin as reference standard (Bradford, 1976).

##### Ectonucleotidase Activity of Islet Homogenate

In order to determine the effect of compounds on ectonucleotidase activity in mice islets, standard curve based procedure was used where KH_2_PO_4_ was used as a standard. A stock solution of KH_2_PO_4_ (1 mM) was prepared in assay buffer that was used to prepare the working solutions of lower concentrations (0–100 µm). A standard curve was generated by incubating increasing concentration of KH_2_PO_4_ with buffer and malachite green reagent.

The assay was carried out in tris-buffer (pH 7.4) containing tris-HCl (50 mM) and CaCl_2_ (5 mM), as previously described (Lavoie et al., 2010). Compounds were dissolved in DMSO (10%) and tested at a concentration of 100 µM. The assay was started by adding 10 µl of test compound solution to 56 µl of assay buffer, followed by the addition of 6 µl of homogenate (i.e., 3 µg of protein). The mixture was incubated at 37°C for 10 min and then 10 µl of ATP (100 µM) was added to start the reaction. Reaction mixture was again placed in incubator at 37°C for 15 min and then reaction was stopped by adding 100 µl of trichloroacetic acid (10%). These samples were placed on ice for 15 min. Finally, samples were mixed with malachite green reagent in an appropriate proportion and released Pi was determined by using standard curve. Moreover, test compounds were tested at different concentrations and dose response curves were generated indicating the amount of Pi released at corresponding dose. All the experiments were carried out in triplicate and enzyme activity was expressed as nm of Pi/min/mg of protein.

#### Real Time Quantitative Polymerase Chain Reaction

##### Treatment of Islets With Test Compound

Freshly isolated mice islets were incubated in KRBB at 37°C with glucose (3 mM). After 45 min, these islets were treated with test compounds in KRBB at 37°C for 3 h, in the presence of stimulatory glucose concentration. Once the incubation was complete, supernatant was removed carefully, and RNA was extracted from these islets.

##### Total RNA Extraction and Real Time Quantitative Polymerase Chain Reaction

Total RNA was extracted using TRIzol reagent, according to manufacturer’s instruction. The concentration of isolated RNA was quantified by using Lvis plate method (FLUOstar Omega, BMG Labtech, Ortenberg, Germany). This RNA was then reverse transcribed to cDNA in a total reaction volume of 20 µl. Briefly, a reaction mixture containing RNA (1 µg) and oligo-dT (20 μM, 1 µl) was prepared and volume was adjusted to 12 µl with nuclease free water. The prepared mixture was incubated at 65°C for 5 min and then immediately chilled on ice. Afterward, 8 µl of master mix containing 5X reaction buffer (4 µl), rnase inhibitor (1 µl), dNTPs mix (10 mM, 2 µl) and reverse transcriptase (1 µl) was added to above sample. After gentle pipetting, sample was run under following conditions: 25°C for 5 min, 42°C for 70 min and 70°C for 5 min. Once the cDNA was synthesized, real time quantitative PCR was conducted by using PikoReal 96 Real-Time PCR system (Thermo Scientific, Vantaa, Finland). For this purpose, a master mixture containing cDNA (1 µl), forward primer (0.25 µl), reverse primer (0.25 µl) and SYBER green master mix (10 µl) was prepared and total volume was adjusted to 20 µl with nuclease free water. Subsequently, thermal cycling conditions were set as follows: 95°C for 10 min and then 40 cycles at 95°C for 15 s and 55°C for 1 min. Relative expression was determined by normalization to β actin mRNA by ΔΔC_T_ method.

### Molecular Docking Studies

Molecular docking analysis was carried out on the basis of results obtained in enzyme kinetics. Hence molecular docking was carried out for the most potent inhibitor of *h*-NTPDase2 (**8j**), *h*-NTPDase3 (**8c**) and *h*-NTPDase8 which showed a competitive mode of inhibition. Compound **8e** revealed a non-competitive mechanism of inhibition against *h*-NTPDase1, therefore its docking was not performed. Since the crystal structures of human NTPDases are not yet available from the Protein Data Bank, their homology models were built according to our previously reported method (Iqbal and Shah, 2018). Docking studies were carried out using BioSolveIT’s LeadIT software^1^. Out of the top ten docked conformations obtained for each inhibitor, the one with the most favorable binding free energy was selected, using HYDE functionality of software. SeeSAR analysis of the inhibitors was also performed by BioSolveIT’s SeeSAR^2^.

### Statistical Analysis

The data was presented as mean ± SEM and statistical analysis was performed using PRISM 5.0 (GraphPad, San Diego, United States). Statistical tests were performed by using student t test and one-way ANOVA. A *p* value < 0.05 was considered significant.

## Results

### Chemistry

**SCHEME 1:**
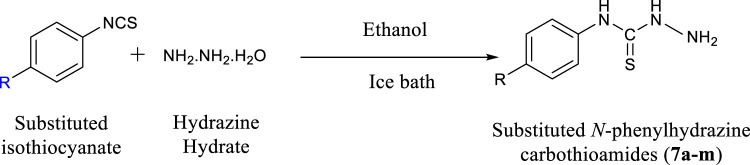
Synthesis of substituted *N*-phenylhydrazine carbothioamides (**7a-m**).

A series of 13 hydrazine carbothioamide derivatives (**8a-m**) was synthesized as potential NTPDase inhibitors. Initially, substituted phenylhydrazine carbothioamides (**7a**-**m**) were prepared by dropwise addition of appropriate isothiocyanate to excess of hydrazine hydrate in ethanol as shown in Scheme 1. The target compounds (**8a-m**) were synthesized by refluxing the synthesized intermediates (**7a-m**) with isatin for 4 to 5 h (Table 1). After the reaction was completed, gradual evaporation of solvent at room temperature resulted in precipitate formation. These precipitates were filtered, washed with ethanol and then dried. The synthesized compounds (**8a-m**) were identified by different spectroscopic techniques including IR, LC/ESI-MS,^1^H-NMR and ^13^C-NMR.

**TABLE 1:** Synthesis of compounds (**8a-pm**).

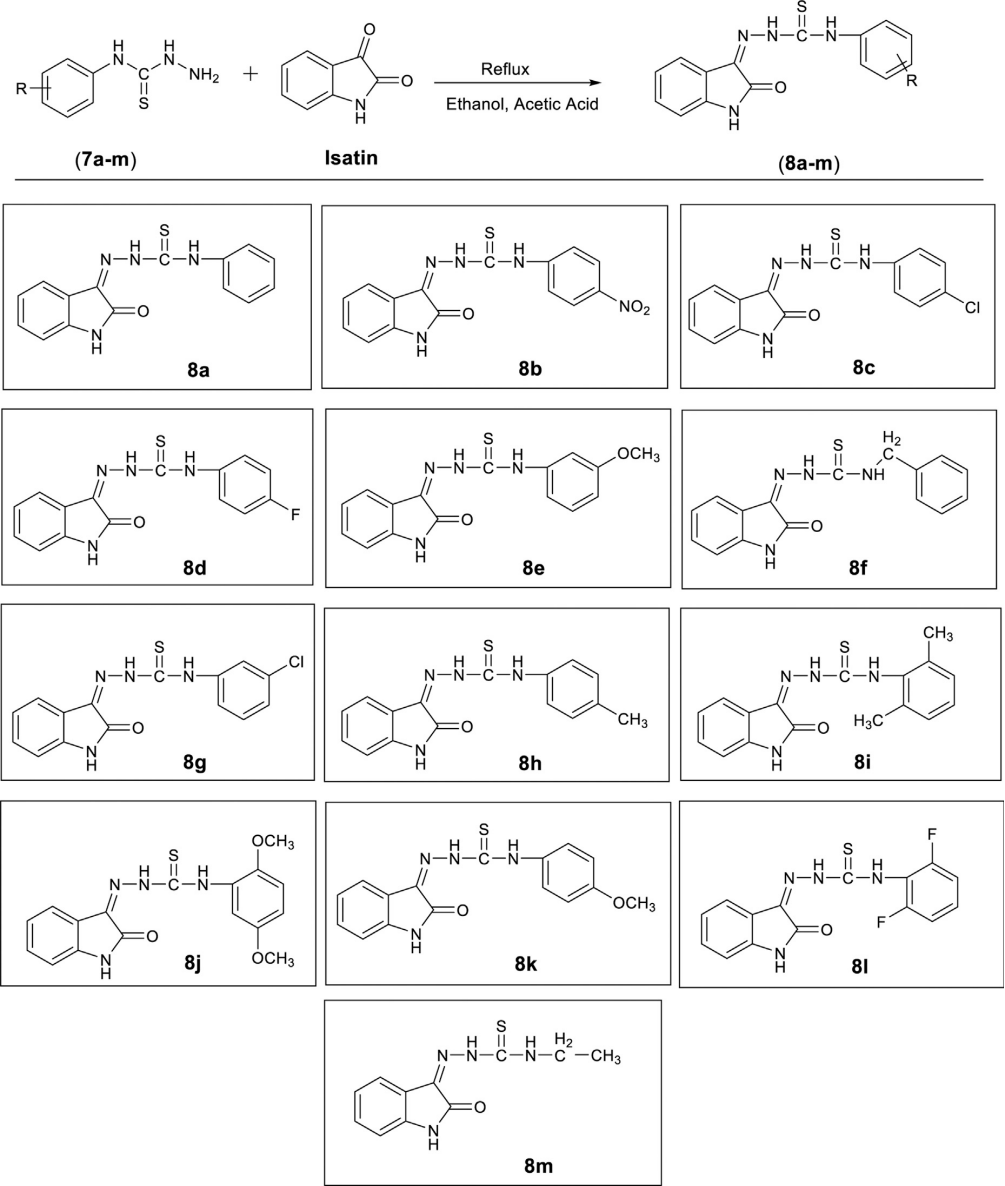

The IR spectra of synthesized compounds showed absorption bands in the region of 3,315–3,130 cm^−1^, resulting from NH stretching of indole and thioamide function. The absorption bands in the region of 1,698–1,686 (cm^−1^) and 1,623–1,591 (cm^−1^) can be assigned to the presence of C = O and C = N, respectively. Likewise, bands in the range of 1,560–1,541 (cm^−1^) and 1,174–1,158 (cm^−1^) reflect the presence of N-H and C = S, respectively. In case of ^1^H-NMR, spectra showed three characteristic peaks (singlet) of NH in the range of δ 9.82–11.12 ppm, 11.22–11.31 ppm, 13.01–12.66 ppm, assigned to CS-NH, indole NH and –N-NH, respectively, confirming the synthesis of desired compounds. All the aromatic protons appeared (as expected) in the region of δ 8.29–6.82 ppm. The ^13^C spectra showed thioamide carbon peak in the range δ 179.09–175.64 ppm, while carbonyl and imine carbon appeared in the range δ 163.20–163.09 ppm and δ 145.12–142.71 ppm, respectively (Pervez et al., 2009; Ali et al., 2014). Mass spectra of synthesized compound was recorded in positive mode, compounds showed molecular ions of varying intensity indicating molecular weights of the compounds. The information regarding _1_H and _13_C spectra of synthesized compounds was presented in Supplementary Material.

A typical structure of a hydrazine carbothioamide showed characteristic signals in ID and 2D NMR spectra. The broad signals for NH groups appeared at *δ*_H_ 12.77, 11.24, and 10.62 ppm, respectively. The doublets of H-4 and H-7 appeared at *δ*_H_ 7.48 and 6.94 ppm, respectively. The carbon signals for *δ*_C_ C = O and C-S appeared at 163.1 and 171.4 ppm. The other characteristics signals of CH, C and CH_3_ groups confirm the structure of compound **8i** and are presented in Table 2.

**TABLE 2:** ^1^H NMR data of compound **8i**.

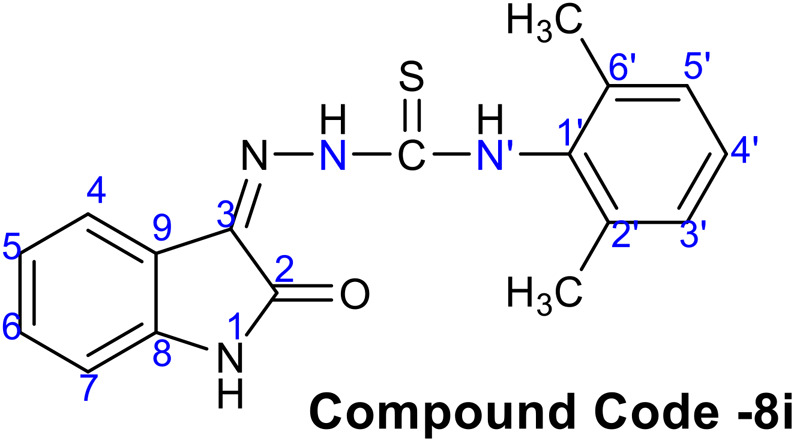				
Atom No (N or C)	*δ*_H_	*δ*_C_	HSQC	HBMC
N*H*	12.77 (brs, 1H)	—	—	—
Nˊ*H*	11.24 (brs, 1H)	—	—	—
N*H*	10.62 (brs, 1H)	—	—	—
C*H*-4	7.48 (d, *J* = 7.5 Hz, 1H)	121.7	C-4	C-6, C-8
C*H*-6	7.37 (t, *J* = 7.5 Hz, 1H)	131.8	C-6	C-4, C-8
C*H*-3ˊ/4ˊ/5ˊ	7.20–7.46 (m, 3H)	128.3/127.9	C-3ˊ/4ˊ/5ˊ	C-1ˊ/5ˊ, C-2ˊ/6ˊ, C-1ˊ/3ˊ
C*H*-5	7.09 (t, *J* = 7.5 Hz)	122.8	C-5	C-7, C-9
C*H*-7	6.94 (d, *J* = 7.8 Hz)	111.5	C-7	C-5, C-9
Ar(C*H*_3_)_2_	2.21 (s)	18.3	Ar(C*H*_3_)_2_	C-1ˊ, C-3ˊ, C-5ˊ
C-1ˊ		136.8	—	—
C-2ˊ		136.5	—	—
C-5ˊ		136.5	—	—
C-2		163.1	—	—
C-3		132.6	—	—
C-8		142.9	—	—
C-9		120.5	—	—
C=S		171.4	—	—

### Effect of Compounds on Recombinant *h*-NTPDases

The synthesized compounds (**8a**-**m**) were investigated for their ability to inhibit *h*-NTPDases using the malachite green assay. Recombinant *h*-NTPDase1, -2, -3 and -8 were employed and initial screening was carried out at an initial concentration of 100 µM. In order to compare species difference, compounds were also tested against mouse NTPDases (*m*-NTPDases), including *m*-NTPDase1, -2, -3 and -8. Dose response curves were generated for compounds showing >50% inhibition of any isoform of enzyme. The inhibitory activity of these compounds against human and mouse NTPDases is presented in Tables 3, 4, respectively.

**TABLE 3:** Human NTPDase inhibitory data for compounds **(8a-m)**.

Code	NTPDase1	NTPDase2	NTPDase3	NTPDase8	NTPDase1	NTPDase2	NTPDase3	NTPDase8
	IC_50_ (µM) ± SEM^a^ or %inhibition at 100 µM^b^				Ki (µM)			
**8a**	45%^b^	24%^b^	20%^b^	24%^b^	—	—	—	—
**8b**	0.29 ± 0.02^a^	19%^b^	20%^b^	24%^b^	0.04	—	—	—
**8c**	0.23 ± 0.01^a^	46%^b^	0.19 ± 0.02^a^	0.24 ± 0.02^a^	0.03	—	0.08	0.11
**8d**	49%^b^	48%^b^	46%^b^	33%^b^	—	—	—	—
**8e**	0.15 ± 0.009^a^	34%^b^	26%^b^	21%^b^	0.15	—	—	—
**8f**	0.24 ± 0.01^a^	38%^b^	27%^b^	31%^b^	0.03	—	—	—
**8g**	28%^b^	29%^b^	17%^b^	7%^b^	—	—	—	—
**8h**	49%^b^	17%^b^	26%^b^	22%^b^	—	—	—	—
**8i**	1.63 ± 0.15^a^	0.41 ± 0.03^a^	25%^b^	17%^b^	0.24	0.17	—	—
**8j**	0.15 ± 0.01^a^	0.11 ± 0.08^a^	34%^b^	19%^b^	0.02	0.04	—	—
**8k**	44%^b^	0.16 ± 0.01^a^	19%^b^	46%^b^	—	0.07	—	—
**8l**	0.30 ± 0.03^a^	45%^b^	22%^b^	32%^b^	0.04	—	—	—
**8m**	2.60 ± 0.02^a^	42%^b^	0.38 ± 0.03^a^	38%^b^	0.38	—	0.16	—
**Suramin** ^**3**^	16.1 ± 1.02^a^	24.1 ± 3.01^a^	4.31 ± 0.41^a^	>100^a^	2.33	9.92	1.85	—

^a^IC_50_ values are presented as mean ± SEM of three independent experiments.

^b^Percent inhibition determined at 100 μM.

**TABLE 4:** Mouse NTPDase inhibitory data for compounds **(8a-m)**.

Code	NTPDase1	NTPDase2	NTPDase3	NTPDase8	NTPDase1	NTPDase2	NTPDase3	NTPDase8
	IC_50_ (µM) ± SEM^a^ or %inhibition at 100 µM^b^				Ki (µM)			
**8a**	38%^b^	42%^b^	31%^b^	28%^b^	—	—	—	—
**8b**	14%^b^	31%^b^	28%^b^	30%^b^	—	—	—	—
**8c**	28%^b^	36%^b^	27%^b^	36%^b^	—	—	—	—
**8d**	41%^b^	27%^b^	29%^b^	1.45 ± 0.12^a^	—	—	—	0.17
**8e**	0.60 ± 0.03^a^	29%^b^	0.70 ± 0.05^a^	3.80 ± 0.21^a^	0.006	—	0.07	0.44
**8f**	4.14 ± 0.13^a^	34%^b^	32%^b^	38%^b^	0.04	—	—	—
**8g**	48%^b^	36%^b^	27%^b^	36%^b^	—	—	—	—
**8h**	29%^b^	31%^b^	31%^b^	28%^b^	—	—	—	—
**8i**	20%^a^	0.15 ± 0.01^a^	32%^b^	26%^b^	—	0.04	—	—
**8j**	24%^a^	27%^b^	30%^b^	32%^b^	—	—	—	—
**8k**	42%^b^	29%^b^	36%^b^	24%^b^	—	—	—	—
**8l**	3.22 ± 0.19^a^	0.20 ± 0.01^a^	0.32 ± 0.04^a^	13%^b^	0.03	0.05	0.03	—
**8m**	3.60 ± 0.02^a^	1.50 ± 0.11^a^	2.41 ± 0.15^a^	35%^b^	0.04	0.41	0.24	—
**Suramin** ^**4**^	>100^a^	21.0 ± 2.0^a^	31.0 ± 2.0^a^	>100^a^	—	5.67	3.07	—

^a^IC_50_ values are presented as mean ± SEM of three independent experiments.

^b^Percent inhibition determined at 100 μM

#### Structure Activity Relationship

Among thirteen derivatives, eight compounds inhibited *h*-NTPDases to a variable extent, however *h*-NTPDase1 was more susceptible as compared to other isozymes. Compound **8e** (IC_50_ = 0.15 ± 0.009 µM) and **8j** (IC_50_ = 0.15 ± 0.01 µM) revealed the most promising activity against *h*-NTPDase1. A structural comparison of these inhibitors showed that both compounds contained –OCH_3_ group attached to phenyl ring, however, **8e** was mono substituted with –OCH_3_ attached to phenyl ring whereas **8j** was substituted with two –OCH_3_ groups. Hence, excellent activity of **8e** could be due this –OCH_3_ group and incorporation of two –OCH_3_ groups (**8j**) resulted in retention of activity.

Likewise, compounds containing –Cl (**8c**, IC_50_ = 0.23 ± 0.01 µM), –NO_2_ (**8b**, IC_50_ = 0.29 ± 0.02 µM) and –(F)_2_ (**8l**, IC_50_ = 0.30 ± 0.03 µM) also showed promising activities. The lowest activity in the series was shown by compounds substituted with an alkyl group as indicated by IC_50_ values of **8i** (IC_50_ = 1.63 ± 0.15 µM) and **8m** (IC_50_ = 2.60 ± 0.02 µM), respectively.

The data suggest that position of substitution also plays an important role. For example, compound **8c** containing 4-Cl substitution showed remarkable activity (IC_50_ = 0.23 ± 0.01 µM); whereas, introduction of 3-Cl resulted in reduced activity of **8g** (28%). Similarly, position of substituent also contributed toward the activity of **8e** (containing 4-OCH_3_ group) since introduction of 3- OCH_3_ group lead to decreased activity of **8k** (44%).

Moreover, mono-substitution and di-substitution was another factor responsible for improved activity of derivatives. In this case, a comparison of **8d** (49.1%) vs. **8l** (IC_50_ = 0.30 ± 0.03 µM) showed that di substitution was more favored than mono substitution. Same pattern of activity was observed for **8h** vs. **8i**. Above all, four compounds i.e. **8b**, **8e**, **8f** and **8l** selectively inhibited *h*-NTPDase1and their %inhibition values for other isozymes was <50%.

In case of *h*-NTPDase2, three compounds could inhibit this isoform and IC_50_ values of these compounds were in sub-micro molar range. Compounds **8j** (IC_50_ = 0.11 ± 0.08 µM) and **8k** (IC_50_ = 0.16 ± 0.01 µM) shared almost comparable activity, indicating substitution with –OCH_3_ was well tolerated, whether it was a mono substitution or a di substitution.

Compound **8i** (IC_50_ = 0.41 ± 0.03 µM) also possessed good inhibitory activity and it incorporated two –CH_3_ groups as a part of its structure. However, the activity of remaining derivatives was <50%.

There were only two hit compounds (**8c**, **8m**) which showed excellent inhibition of *h*-NTPDase3. A comparison of IC_50_s showed that **8c** was 22 times more active than standard inhibitor i.e. suramin. Structure of **8c** contained 4-Cl attached to it and this activity appeared position dependent since introduction of 3-Cl resulted in loss of activity. Another inhibitor of *h*-NTPDase3 was **8m** and it also showed excellent inhibitory activity (IC_50_ = 0.38 ± 0.03 µM). This compound contained an ethyl chain attached to –NH of thioamide chain, indicating that alkyl chain was well tolerated. However, presence of other substituents like –F, –CH_3_ and –OCH_3_ did not show any remarkable activity toward *h*-NTPDase3.

Only one compound effectively inhibited *h*-NTPDase8 and that was **8c**. The IC_50_ of **8c** (0.24 ± 0.02 µM) was 400 times than that of suramin. However, no other compound could inhibit *h*-NTPDase8 to an appreciable extent.

#### Mechanism of Inhibition of Candidate Compounds

**FIGURE 1:**
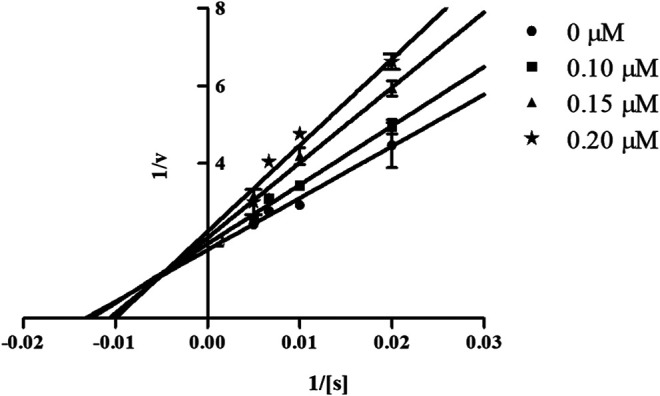
Lineweaver-Burk Plot for *h*-NTPDase1 inhibitor **(8e)**. S represents the substrate concentration (μM) and concentration of inhibitor **(8e)** are 0, 0.10, 0.15, and 0.20 μM.

**FIGURE 2:**
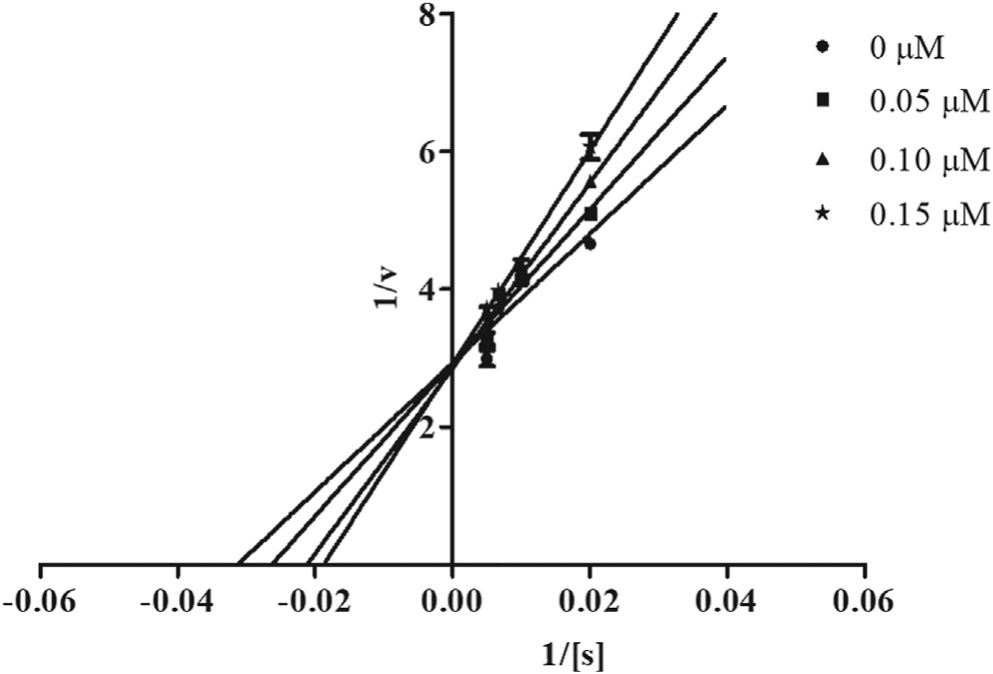
Lineweaver-Burk Plot for *h*-NTPDase2 inhibitor **(8j)** S represents the substrate concentration (μM) and concentration of inhibitor **(8j)** are 0, 0.05, 0.10, and 0.15 μM.

**FIGURE 3:**
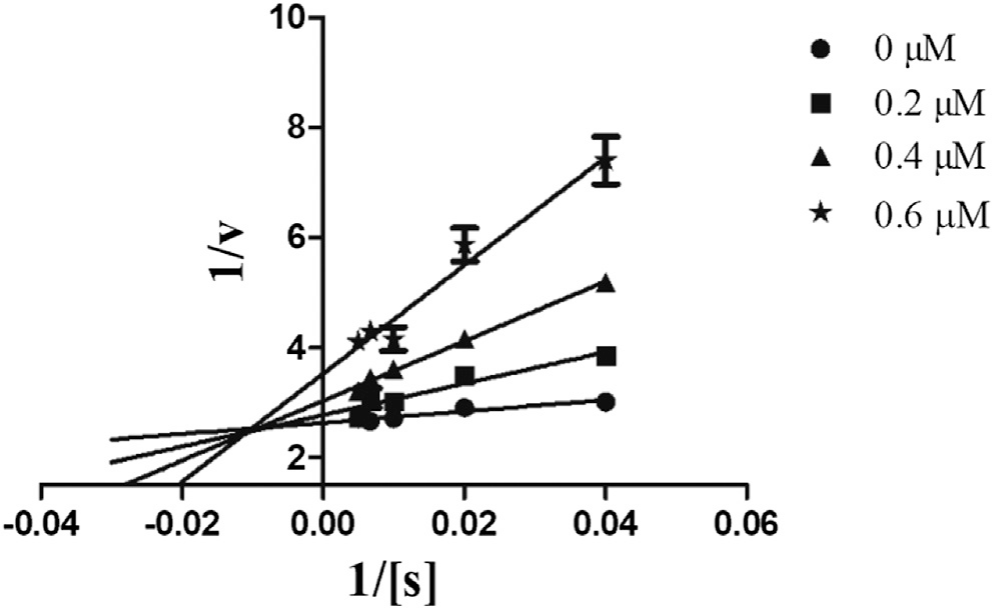
Lineweaver-Burk Plot for *h*-NTPDase3 inhibitor **(8m**). S represents the substrate concentration (μM) and concentration of inhibitor **(8m)** are 0, 0.20, 0.4, and 0.6 μM.

**FIGURE 4:**
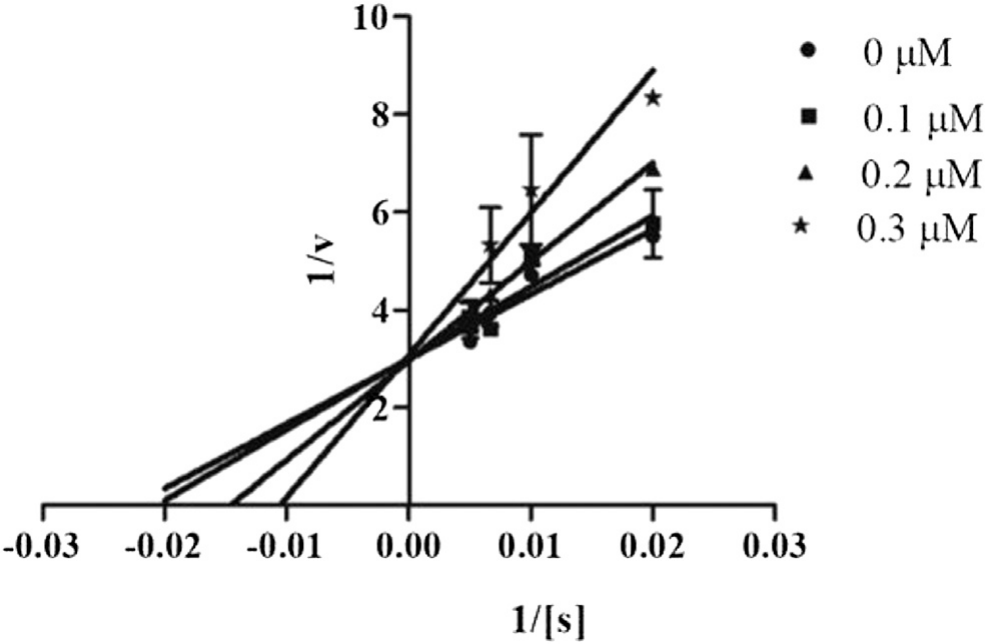
Lineweaver-Burk Plot for *h*-NTPDase8 inhibitor **(8c)**. S represents the substrate concentration (μM) and concentration of inhibitor **(8c)** are 0, 0.10, 0.20, and 0.30 μM.

In order to determine the mechanism of inhibition, kinetic studies of most potent inhibitors were performed against respective isozymes. In this regard, compound **8e** revealed a non-competitive mode of inhibition for *h*-NTPDase1 (Figure 1). However, compound **8j** was found to be a competitive inhibitor of *h*-NTPDase2 since all the four lines (indicating different concentrations of inhibitor) were intersecting at *y*-axis (Figure 2). Compound **8m** showed a non-competitive mode of inhibition for *h*-NTPDase3 (Figure 3) whereas the Lineweaver-Burk plot for *h*-NTPDase8 with compound **8c** exhibited a competitive mechanism of inhibition (Figure 4).

### Effect of NTPDase3 Inhibitors on Insulin Secretion

**FIGURE 5:**
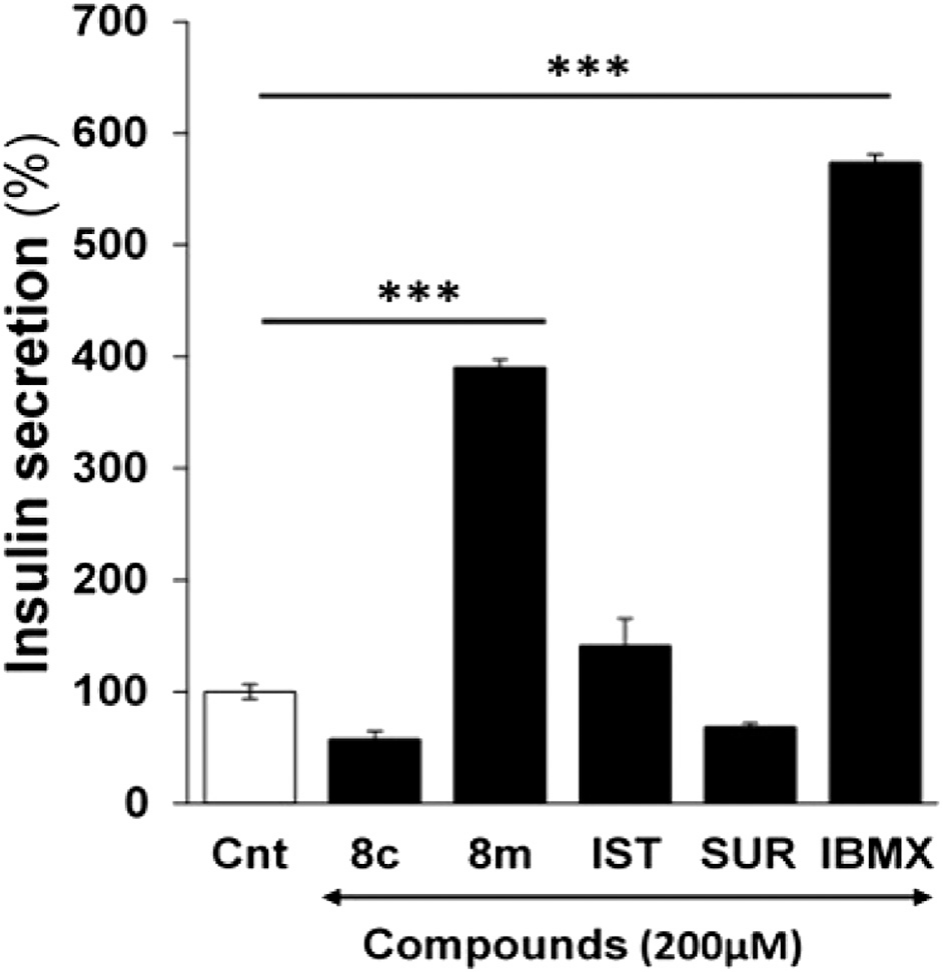
Effect of compound **8c, 8m,** suramin (SUR), isatin (IST) and isobutyl methyl xanthine (IBMX) on glucose stimulated insulin secretion in mice pancreatic islets at 200 µM. Group of size-matched islets were incubated at 37°C for 1 h in KRB buffer with 16.7 mM glucose supplemented with or without test compounds. Values are mean ± S.E.M. from two to three independent experiments. Insulin secretion induced by 16.7 mM Glucose was considered 100%. ***p < 0.0001 vs. none. Cnt, insulin secretion induced by 16.7 mM glucose only.

The ability of *h*-NTPDase3 inhibitors (**8c** and **8m**) to stimulate insulin secretion was determined by using freshly isolated mice islets. It was observed that compound **8m** produced a significant increase in glucose stimulated insulin secretion as compared to control and activity of this compound (**8m)** was comparable to that of isobutyl methyl xanthine (IBMX), used as positive control. The results are shown in Figure 5.

**FIGURE 6:**
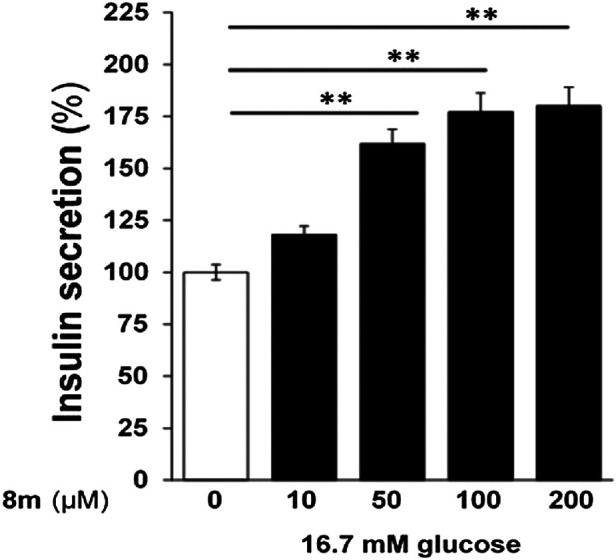
Dose-dependent effect of compound **8m** on insulin secretion. Compound **8m** was employed at doses of 0, 10, 50, 100, and 200 µM concentration supplemented with 16.7 mM glucose. Group of size-matched islets were incubated at 37°C for 1 h in KRB buffer with 16.7 mM glucose supplemented with or without test compounds. Values are mean ± S.E.M. from two to three independent experiments. **p < 0.001.

The compound **8m** was tested at different doses (10–200 µM) and it induced a dose dependent increase in insulin secretion (Figure 6). However, compound **8m** did not produce any significant effect at basal glucose level (3 mM).

Although compound **8c** was the most potent *h*-NTPDase3 inhibitor but it did not show any significant effect on insulin secretion. This discrepancy in the behavior of compound **8c** might be attributed to the species difference. Therefore, we tested all the compound on mouse NTPDases (*m*-NTPDases) and we found that compound **8**c was not an effective inhibitor of *m*-NTPDase3 (Table 4).

Suramin, used as positive control during enzyme inhibition studies, was tested for its insulin secretory activity. Isatin, used as a reactant during synthesis of target compounds, was also investigated for its ability to stimulate insulin secretion. Neither suramin nor isatin had any significant effect on insulin release (Figure 5).

### Effect of 8m on NTPDase3 Activity in Mice Pancreatic Islets

**FIGURE 7:**
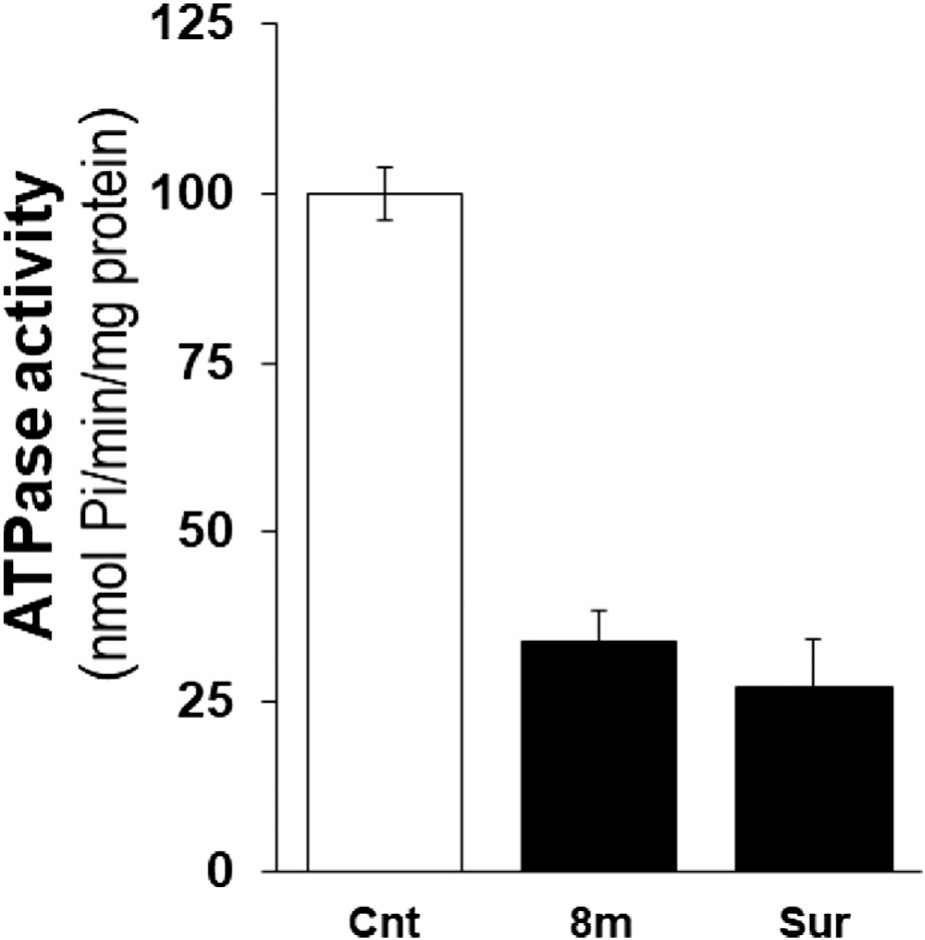
Effect of compound **8m** and suramin on ectonucleotidase activity in islets homogenate using 100 µM ATP as substrate. Enzyme activity is expressed in nmol Pi/min/mg of protein. Enzyme activity without any inhibitor was considered 100%. Treatment of homogenate with compound **8m** and suramin significantly reduced the enzyme activity. Bars represent means ± SEM for three independent experiments. ***p < 0.0001. Cnt (control), enzyme activity without addition of compound, Sur, enzyme activity by suramin.

**FIGURE 8:**
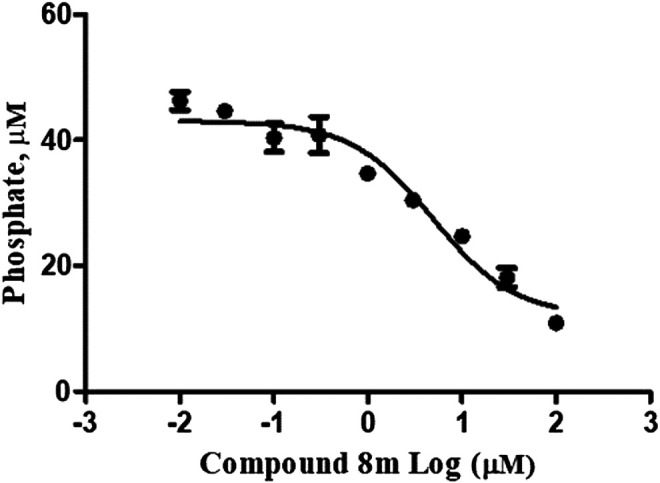
Compound **8m** inhibits the ectonucleotidase activity in islet homogenate, measured as release of Pi from exogenously added ATP at a concentration of 100 µM (n = 3).

Given that compound **8m** was identified as an effective inhibitor of *h*-NTPDase3 as well as a regulator of the glucose-induced insulin secretion, we decided to determine the compound’s effect on ectonucleotidase activity in isolated mice islets. A homogenate was prepared from the isolated islets which was treated with compound and then ectonucleotidase activity was determined by malachite green assay. At a concentration of 100 μM, compound **8m** significantly decreased the ectonucleotidase activity as compared to control. The activity of test compound (**8m**) was comparable to that of suramin, a standard inhibitor of NTPDase (Figure 7). Moreover, a dose response curve was generated for compound **8m** and it was found that compound **8m** produced a dose dependent decrease in enzyme activity (Figure 8).

### Effect of 8m on NTPDase3 mRNA by Real Time Quantitative Polymerase Chain Reaction

To determine if the reduction in ectonucleotidase activity by compound **8m** in mice pancreatic islets was an outcome of downregulation of NTPDase3 mRNA, we analyzed the effect of this compound (**8m**) on NTPDase3 gene expression. Freshly isolated mice islets were incubated with test compound, total RNA was extracted and relative fold change in the NTPDase3 mRNA expression was determined by real time qPCR experiments. The results showed that test compound (**8m**) had no significant effect (*p* > 0.05) on the NTPDase3 gene expression in mice pancreatic islets as compared to control. Moreover, expression level of β-actin (housekeeping gene) was also not affected by compound **8m**.

### Molecular Docking

**FIGURE 9:**
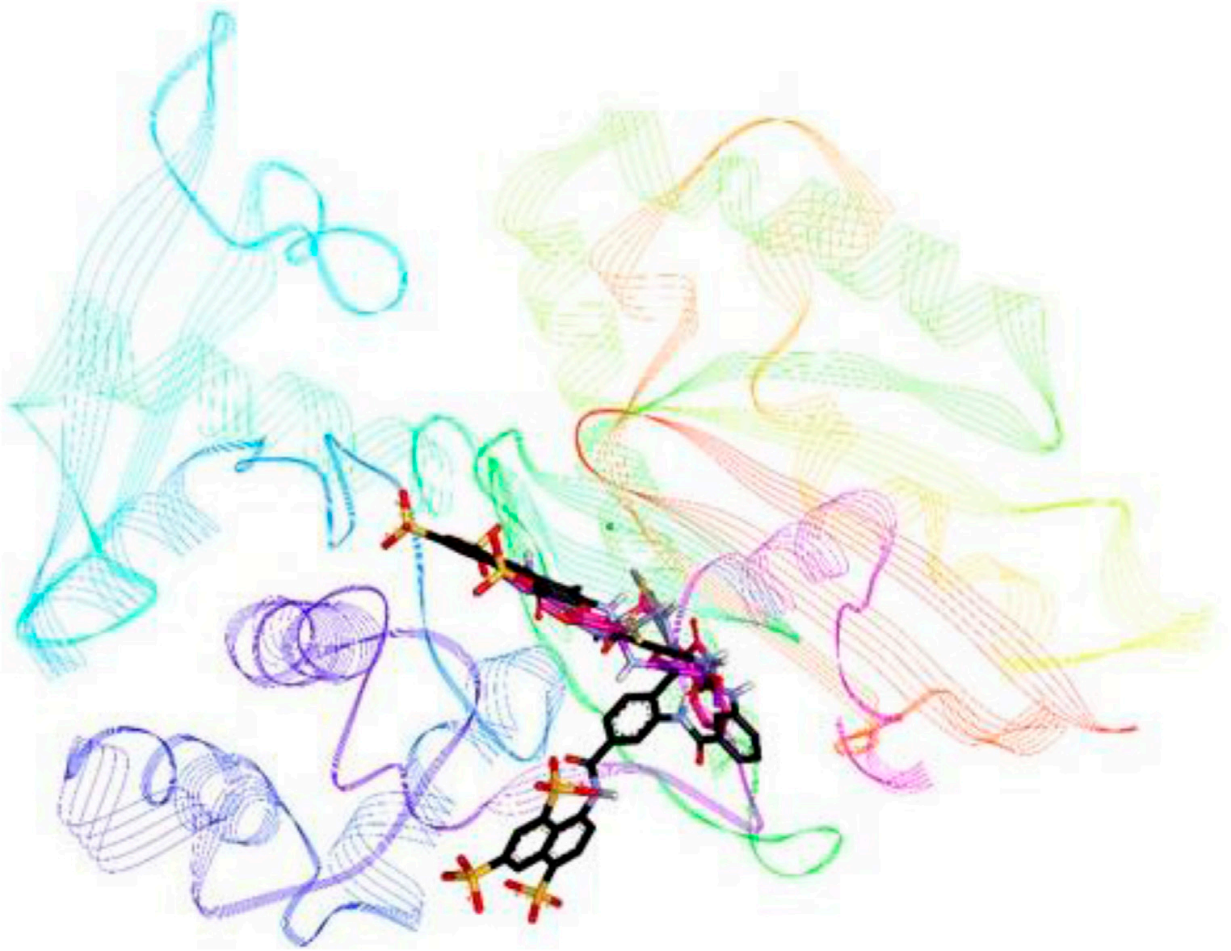
Overlap of docked conformations of compounds **8i** (gray), **8j** (purple) and **8k** (pink) with standard inhibitor suramin (black), the protein backbone is represented in lined ribbon, the calcium ion is shown as green sphere.

Compounds **8i**, **8j** and **8k** were highly active against *h*-NTPDase2 and were therefore docked against this enzyme. For reference, docking of standard inhibitor suramin was also carried out. All compounds were found to bind in the same area of the active site, in the region that is also shared by suramin. Figure 9 shows an overlap of docked conformations of inhibitors **8i**, **8j**, **8k** with suramin.

**FIGURE 10:**
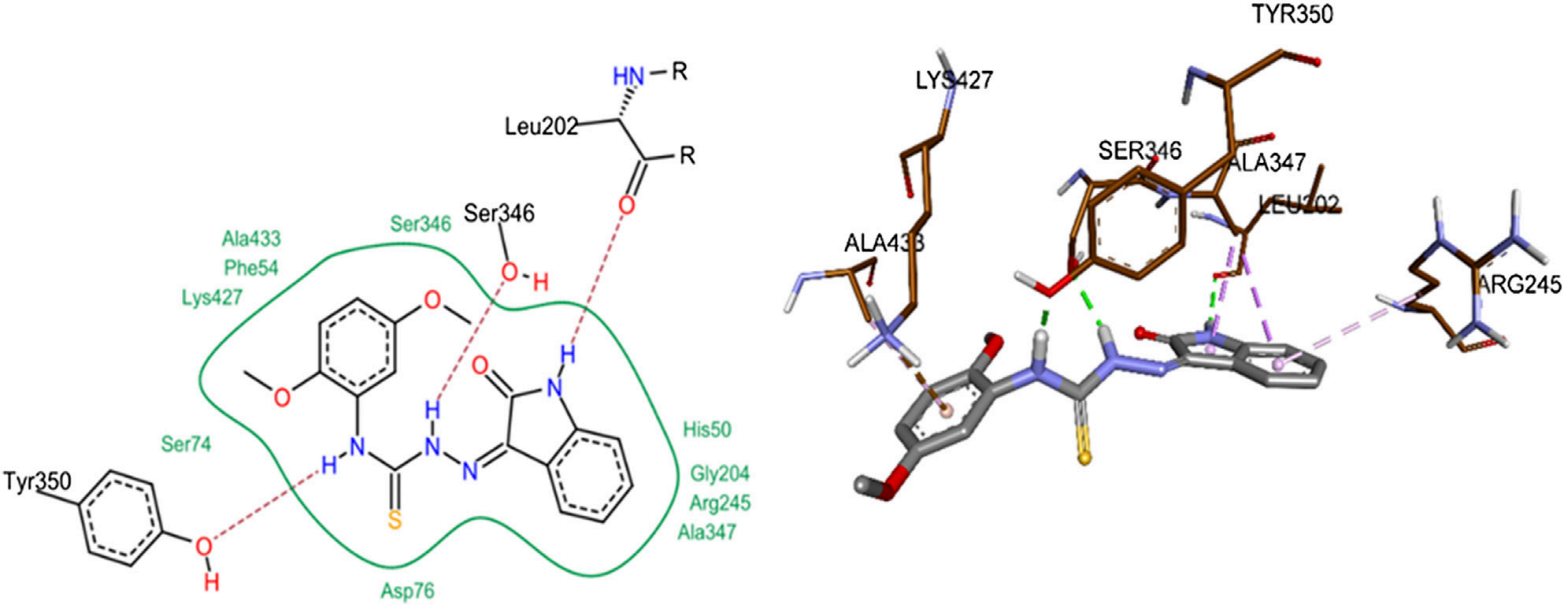
2D **(left)** and 3D **(right)** representation of docked conformations of *h*-NTPDase2 inhibitor **8j**.

The most probable docked conformation of compound **8j**, the most active *h*-NTPDase2 inhibitor, is shown in Figure 10. As can be seen, the NH of the indole ring was making a hydrogen bond with Leu202. Likewise, one of the NH groups of thiourea moiety was making hydrogen bond with Ser346 whereas the other NH group was making hydrogen bond with Tyr350. The 2,5-dimethoxy phenyl ring was making a pi-cation interaction with Ala433, pi-sigma and pi-alkyl interactions were observed between the indole ring and the amino acids Ala347 and Arg245, respectively.

**FIGURE 11:**
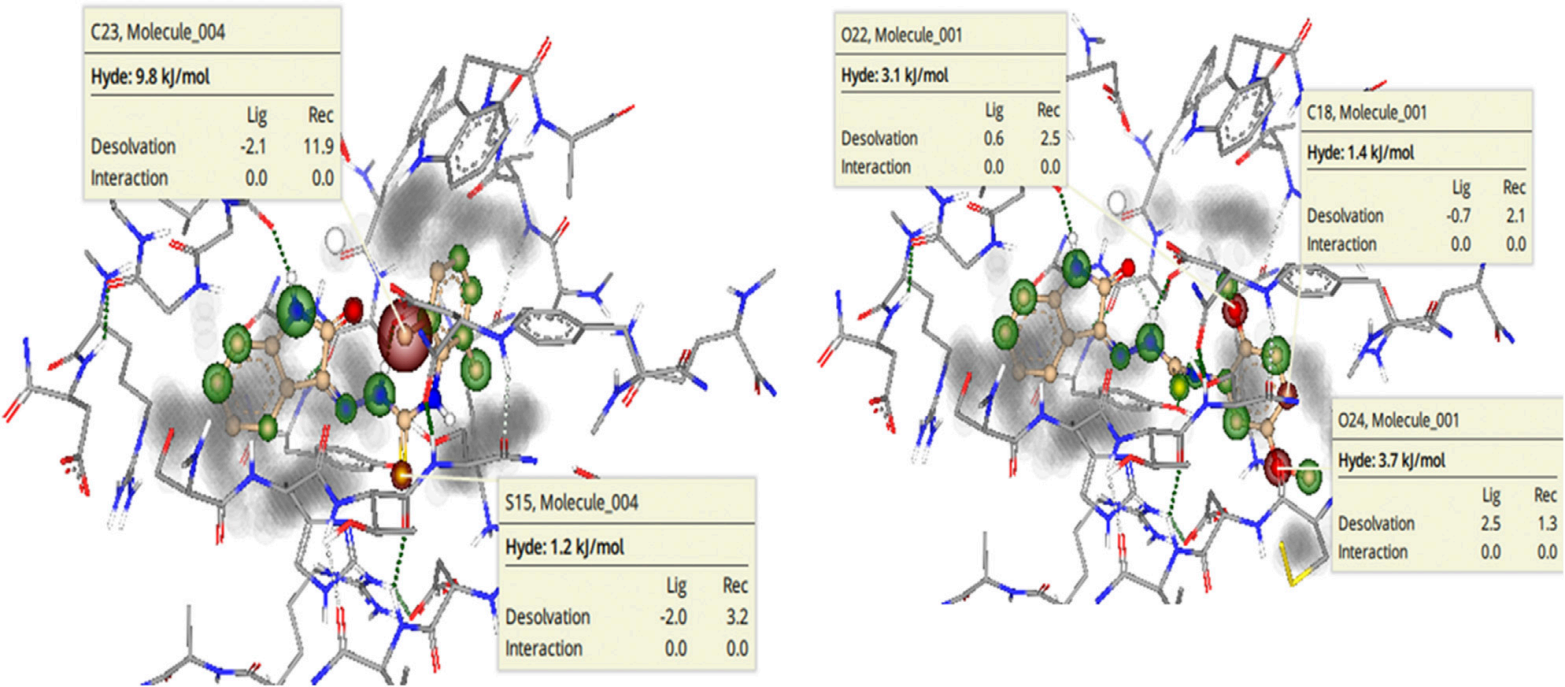
See SAR Analysis (visual representation of contribution of each atom to overall binding affinity) of *h*-NTPDase2 inhibitors **8i** **(left)** and **8j** **(right)**. The structural elements that are contributing favorably to the overall binding affinity are represented with green colored coronas, structural elements that with unfavorable contribution are represented with red colored coronas, neutral elements are not colored. Unoccupied space in the binding site is in gray color.

To further highlight the role of each structural element toward the overall binding affinity SeeSAR analysis was carried out. BioSolveIT’s SeeSAR is a tool that provides visual display of binding affinity. The information thus provided can be very helpful in logically modulating the structures of lead inhibitors, to synthesize even more potent inhibitors. The SeeSAR analysis of most active NTPDase inhibitors **8i** and **8j** is given in Figure 11. The structural features of the compound that are contributing positively to the overall binding affinity are indicated with green coronas; greater the contribution, larger is the size of the corona. Similarly, the structural elements that are not contributing favorably to the overall binding are indicated with red coronas, whereas the structural features with no contribution are not colored. As can be seen, most of the atoms in the molecule **8i** are contributing favorably to overall binding (indicated by green colored coronas), except two structural elements, i) one of the methyl carbon atoms of the 2,6-dimethyl substituted phenyl ring (labeled C23 in Figure 11, 9.8 kJ/mol), and ii) the sulfur atom (S15 in Figure 11, 1.2 kJ/mol) of thiourea (C = S) moiety. These unfavorable contributions are because of high desolvation energy which has not been compensated by any (favorable) non-bonded interaction. Based on the unoccupied space in the binding pocket (indicated in gray), the structure of the molecule can be changed/extended further to provide a more snug fit into the binding pocket. Similarly, for compound **8j**, unfavorable contribution to binding free energy is because of somewhat high desolvation energy of methoxy oxygen atoms (O22 and O24, 3.1 and 3.7 kJ/mol, respectively) and one of the phenyl ring carbon atoms (C18, 1.4 kJ/mol). Replacement of these atoms with some other suitable atoms/group is expected to result in even more active inhibitors.

**FIGURE 12:**
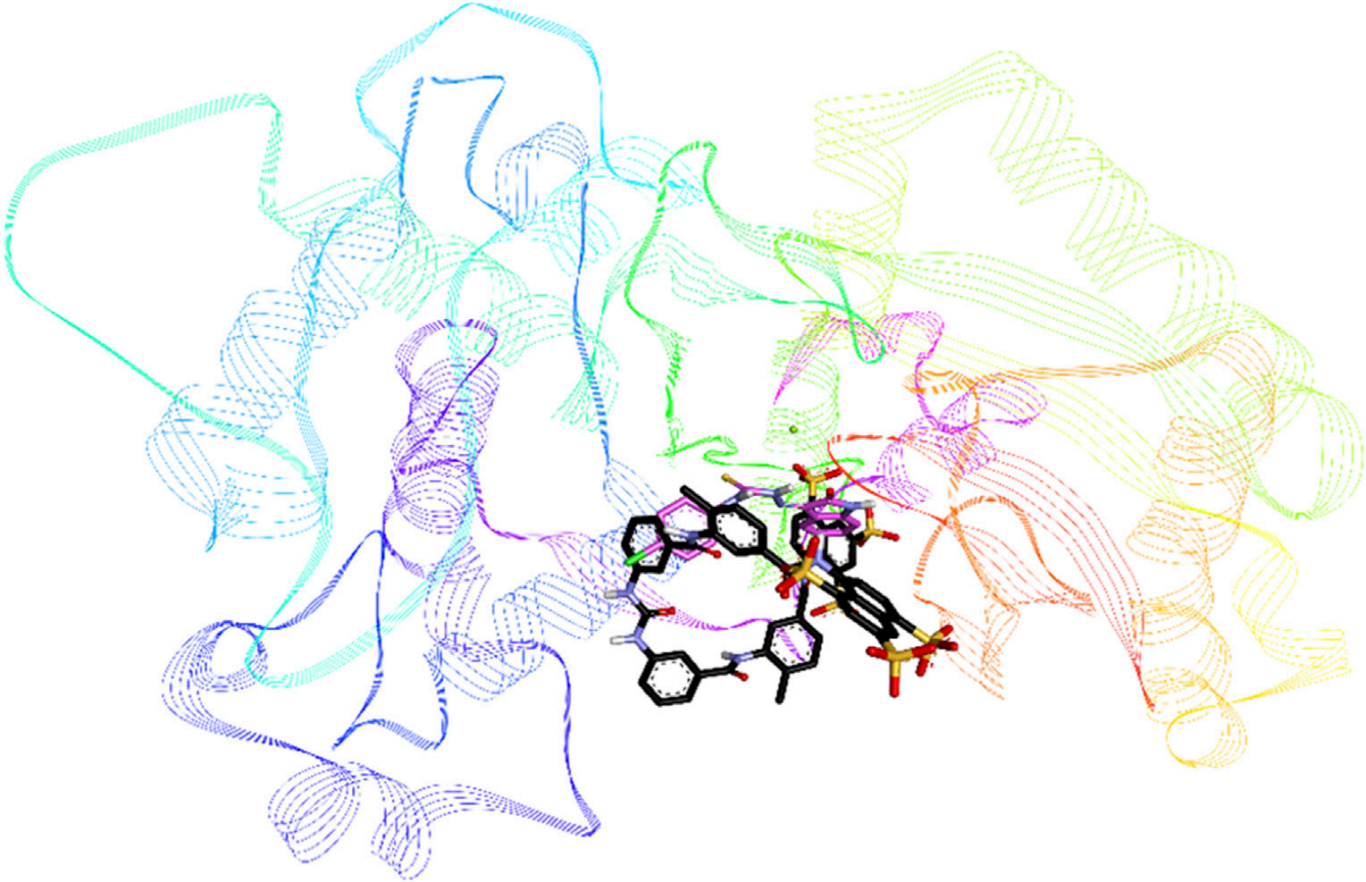
Overlap of docked conformations of *h*-NTPDase3 inhibitor **8c** (gray) and standard inhibitor suramin (black), the protein backbone is represented in lined ribbon, the magnesium ion is shown as a green sphere.

**FIGURE 13:**
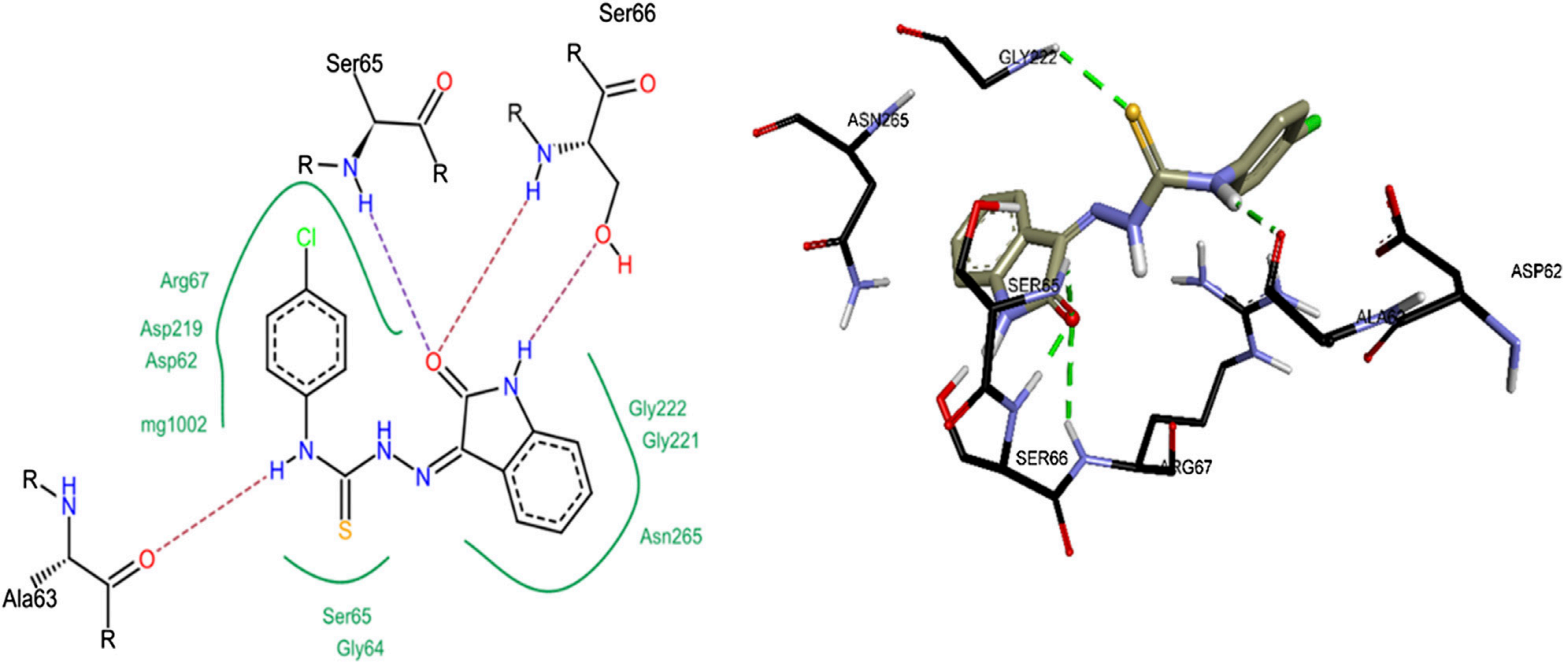
2D **(left)** and 3D **(right)** representation of docked conformations of *h*-NTPDase3 inhibitor **8c**.

Compound **8c** was the most active inhibitor of *h*-NTPDase3 and showed competitive mode of inhibition, hence its docking studies were carried out. For reference, suramin the standard inhibitor, was also docked. Figure 12 shows overlap of docked conformation of **8c** with that of suramin. Docking studies of compound **8c** revealed a number of hydrogen bonded interactions, the indole NH was making a hydrogen bond with Ser66, while the carbonyl oxygen (of indole ring) was within hydrogen bond distance of Ser65 and Ser66. The NH group of thiourea moiety was making hydrogen bond with Ala63 (Figure 13).

**FIGURE 14:**
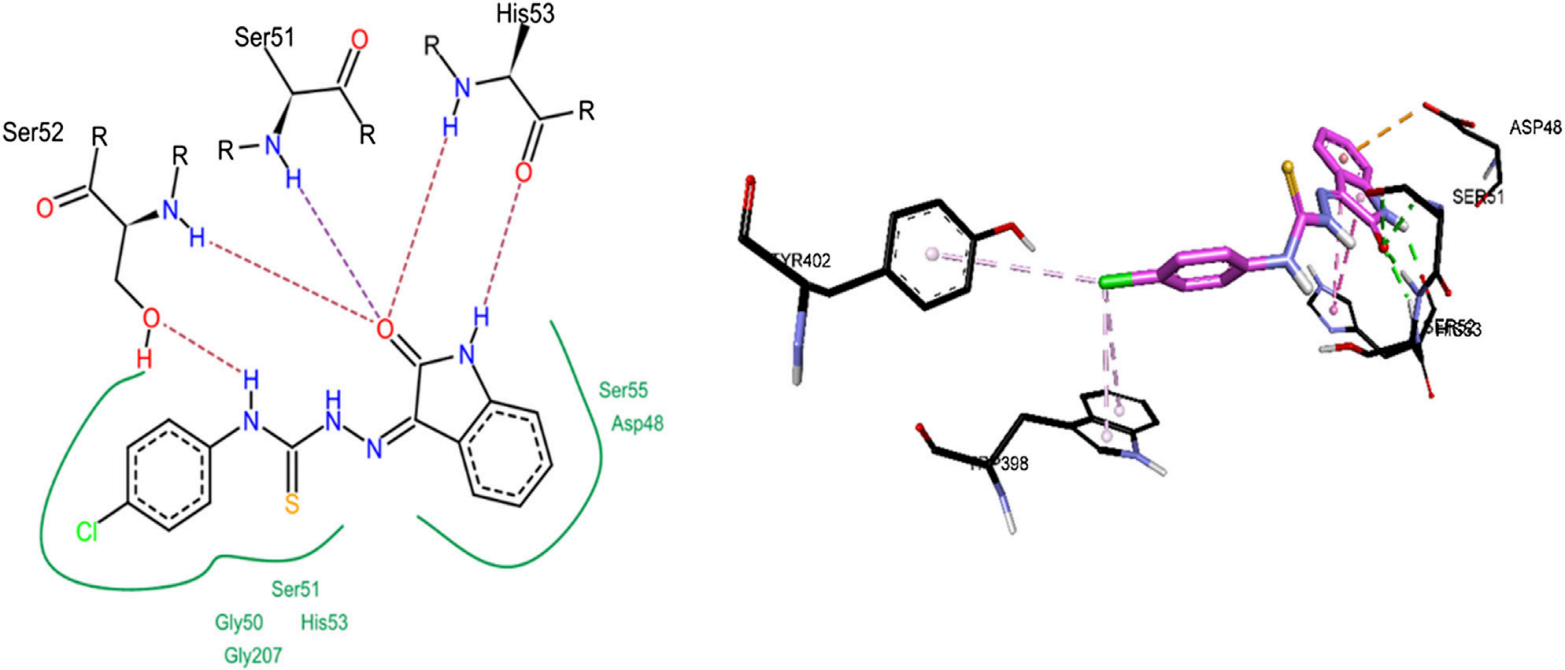
2D **(left)** and 3D **(right)** representation of docked conformations of *h*-NTPDase8 inhibitor **8c**.

Compound **8c** was the only inhibitor of *h*-NTPDase8 and showed competitive mode of inhibition, hence its docking studies were carried out. As shown in Figure 14, compound **8c** revealed a number of hydrogen bonded interactions, the indole NH was making a hydrogen bond with His53, while the carbonyl oxygen (of indole ring) was within hydrogen bond distance of Ser52, Ser51 and His53. The NH group of thiourea moiety was making hydrogen bond with Ser52. Additionally, a pi-pi stacked interaction was also observed between the indole ring and His53. A pi-anion interaction was observed between indole phenyl ring and Asp48.

**FIGURE 15:**
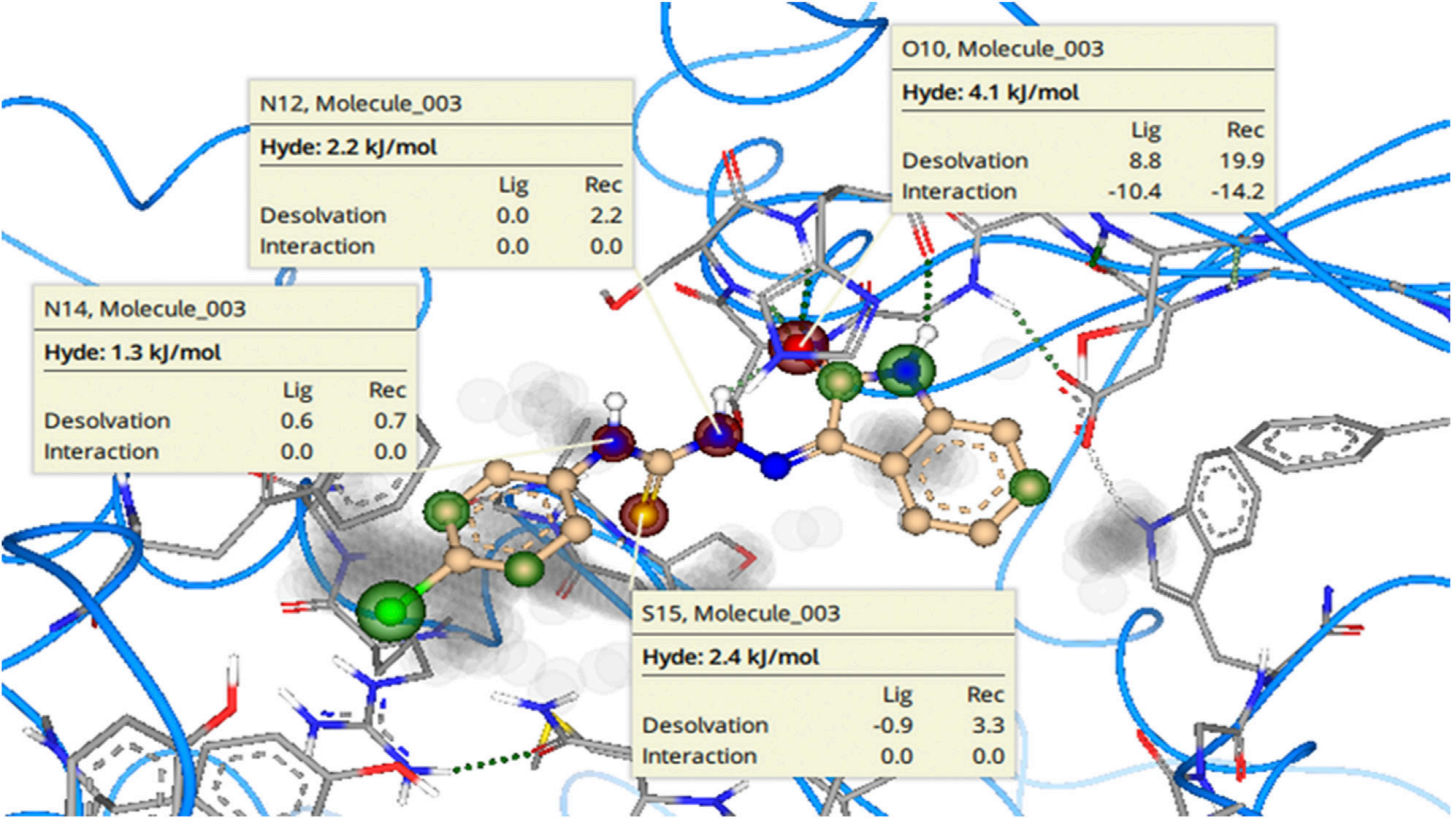
See SAR Analysis (visual representation of contribution of each atom to overall binding affinity) of *h*-NTPDase8 inhibitor **8c**. The structural elements that are contributing favorably to the overall binding affinity are represented with green colored coronas, structural elements that with unfavorable contribution are represented with red colored coronas, neutral elements are not colored. Unoccupied space in the binding site is in gray color.

SeeSAR analysis of *h*-NTPDase8 inhibitor **8c** was also carried out as depicted in Figure 15. The structural elements with unfavorable contribution to the overall binding free energy are indicated in red coronas. These include the nitrogen atoms (N14, 1.3 kJ/mol; N12, 2.2 kJ/mol) and the sulfur atom (S15, 2.4 kJ/mol) of the thiourea moiety. This is because of high desolvation energy. The oxygen atom of the carbonyl group (O10 4.1 kJ/mol) also had high desolvation energy, although it was making a hydrogen bond, yet the compensation is not enough to fully compensate the penalty of (high) desolvation energy. It is suggested that replacement of these atoms with some other suitable atoms/group is expected to result in even more active inhibitors.

A summary of amino acids interacting with NTPDase inhibitors as well as binding energy associated with these inhibitors is presented in Table 5. These computed binding energies were compared with biological data in Table 6, where biological data is presented in the form of IC_50_ and Ki values. The binding energy values were in close agreement with the experimentally determined IC_50_/Ki values. For instance, the most potent *h*-NTPDase2 inhibitor (**8j**, IC_50_ = 0.11 ± 0.08 µM, Ki = 0.04 µM) had the best binding energy of −29 kJ/mol. Similarly, compounds **8c** with the binding energy of −27 kJ/mol was found to be the most potent inhibitor of *h*-NTPDase3 (IC_50_ = 0.19 ± 0.02 µM, Ki = 0.08) and *h*-NTPDase8 (IC_50_ = 0.24 ± 0.02 µM, Ki = 0.11 µM).

**TABLE 5:** Summary of amino acids interacting with NTPDase inhibitors.

NTPDase inhibitors	Binding energy (kJ/mol)	Interacting amino acid residues	
		H-bond donors	H-bond acceptors
**8i** (NTPDase2)	−26	Nil	Leu202 (C=O), Ser346 (−OH), Ser52 (−OH)
**8j** (NTPDase2)	−29	Nil	Tyr350 (−OH), Ser346 (−OH), Leu202 (C=O)
**8k** (NTPDase2)	−29	Arg392 (NH(NH_2_)^+^NH_2_)	Ser346 (−OH), Ser52 (−OH)
**8c** (NTPDase3)	−27	Ser65 (−NH), Ser66 (−NH)	Ala63 (C=O), Ser66 (−OH)
**8c** (NTPDase8)	−27	Ser51 (−NH), Ser52 (−NH), His53 (−NH)	His53 (C=O), Ser52 (−OH)

**TABLE 6:** Comparison of IC_50_, Ki and binding energy of the most potent NTPDase inhibitors.

*h*-NTPDase inhibitor	IC_50_ (µM)	Ki (µM)	Binding energy (KJ/mol)
**8i** (NTPDase2)	0.41 ± 0.03	0.17	−26
**8j** (NTPDase2)	0.11 ± 0.08	0.04	−29
**8k** (NTPDase2)	0.16 ± 0.01	0.07	−29
**8c** (NTPDase3)	0.19 ± 0.02	0.08	−27
**8c** (NTPDase8)	0.24 ± 0.02	0.11	−27

## Discussion

In this study, we have synthesized and evaluated a series of oxoindolin hydrazine carbothioamide derivatives as potential NTPDase inhibitors. Most of the compounds appeared to be more potent inhibitors of *h*-NTPDase1 than other NTPDases. Compound **8e** and **8j** were the most potent *h*-NTPDase1 inhibitors whereas compound **8j** was also the most potent inhibitor of *h*-NTPDase2. Both of these compounds contained an –OCH_3_ group as a part of their structure, thus implying the importance of this –OCH_3_ for *h*-NTPDase1 and -2 inhibitors. On the other hand, compound **8c** was found to be potent dual inhibitor of *h*-NTPDase3 and -8. Interestingly, compound **8b**, **8e**, **8f**, and **8l** were identified as selective inhibitors of *h*-NTPDase1 whereas compound **8k** selectively inhibited the *h*-NTPDase2. Furthermore, compound **8e**, **8j**, **8c**, and **8m** were studied in detail to establish their mechanism of inhibition. Compound **8e** inhibited *h*-NTPDase1 in a non-competitive manner whereas compound **8j** and **8c** revealed a competitive mode of inhibition against *h*-NTPDase2 and -8, respectively. Kinetics studies of compound **8m** revealed that it was inhibiting the *h*-NTPDase3 in a non-competitive manner.

In literature, different classes of compounds have been synthesized as NTPDase inhibitors, including nucleotide analogues, polyoxometalates (POMs) and anthraquinone derivatives. Among them, POMs showed best inhibitory activity with Ki value as low as 0.0038 µM (Lee et al., 2015) whereas 8-Bus-ATP (nucleotide analogue) was found to be a selective inhibitor of *h*-NTPDase1 (Ki = 0.8 ± 0.2 µM) (Lecka et al., 2013). The Ki values of our compounds against *h*-NTPDases were found to be in the range of 0.02–0.38 µM. Thus, our compounds tend to be more potent than 8-Bus-ATP but their activity is low as compared to POMs. A recent study (Baqi et al., 2020) reported synthesis of anthraquinone derivatives as potent *h*-NTPDase inhibitors, showing selectivity against *h*-NTPDase2 and -3. The IC_50_ values of these compounds were found to be within the range of 0.39–15.3 µM, while the IC_50_ values of our compounds were within the range of 0.11–2.60 µM, indicating that our compounds are more active *h*-NTPDase inhibitors than anthraquinone derivatives.

NTPDase3 is the most prominent isoform in pancreatic islets where it has been reported to regulate the insulin secretion (Bartley et al., 2019; Saunders et al., 2019). Therefore, NTPDase3 inhibitors i.e. **8c** and **8m** were evaluated for their effects on insulin secretion in mice islets. Compound **8m** produced a dose dependent increase in insulin release, in the presence of high glucose concentration. At basal glucose level, compound **8m** has no effect on insulin secretion since glucose is the actual initiator of insulin release, whereas ATP can stimulate the insulin secretion only in the presence of high glucose concentration (Cieślak and Cieślak, 2017). On the other hand, compound **8c** had no significant effect on insulin secretion due to species difference, as the screening of compounds on *m*-NTPDases showed that compound **8c** exhibited < 50% inhibition against *m*-NTPDase3. Although compound **8m** was showing good inhibitory activity against *m*-NTPDase1, -2 and -3, it was the *m*-NTPDase3 inhibition that potentiated the insulin secretion since NTPDase3 is the predominant isoform expressed in mice islets. In this context, a study had reported that NTPDase3 was the most abundant isoform in mouse and human islets whereas least amounts of NTPDase1 and -2 were detected. They used a non-selective NTPDase inhibitor (ARL67156) and established that it was the NTPDase3 inhibition that potentiated insulin secretion. They also performed siRNA experiments and observed that the knockdown of NTPDase3 expression was producing the same effects on insulin secretion as those obtained with ARL67156, thus suggesting that it is NTPDase3 inhibition that is contributing to insulin secretion (Syed et al., 2013).

Furthermore, we also determined the inhibitory effect of compound (**8m**) in mice islets and it produced a significant reduction in ectonucleotidase activity, suggesting that compound (**8m**) is stimulating the insulin secretion via NTPDase3 inhibition. However, inhibition of NTPDase3 (by compound **8m**) was not occurring due to the downregulation of NTPDase3 mRNA since compound (**8m**) had not suppressed the gene expression of NTPDase3. Finally, molecular docking studies and SeeSAR analysis of the most potent inhibitors were also carried out and the calculated binding energies were in accordance with our experimental data. Taken together, our study demonstrates that **8m** is a potent inhibitor of NTPDase3 which is involved in the stimulation of glucose induced insulin secretion without suppressing the NTPDase3 gene.

## Conclusion

In a series of oxoindolin hydrazine carbothioamide derivatives, only two compounds i.e. **8c** (IC_50_ = 0.19 µM ± 0.02) and **8m** (IC_50_ = 0.38 ± 0.03) showed excellent inhibition of *h*-NTPDase3. These inhibitors of *h*-NTPDase3 were investigated for their effects on insulin secretion and only compound **8m** was found as a lead regulator of insulin secretion. Further studies revealed that compound **8m** significantly reduced ectonucleotidase activity in mice pancreatic islets. In consistence with our *in vitro* data, docking studies displayed strong binding interaction of potent inhibitors within the active site of respective enzyme. In conclusion, we report compound **8m** as a potent inhibitor of *h*-NTPDase3, stimulating the glucose induced insulin secretion. Further study is needed to investigate **8m** as a potential drug candidate.

## Data Availability Statement

The raw data supporting the conclusions of this article will be made available by the authors, without undue reservation, to any qualified researcher.

## Ethics Statement

This study was reviewed and approved by Research Ethics Committee, Department of Pharmacy, COMSATS University Islamabad, Abbottabad Campus.

## Author Contributions

SA carried out synthesis and enzyme assays, Mal-R Designed Synthesis of the derivatives, AH supervised the effect on glucose stimulated insulin secretion, JP and JS expressed the enzymes used in the study. JI designed the project and supervised the biological experiments.

## Conflict of Interest

The authors declare that the research was conducted in the absence of any commercial or financial relationships that could be construed as a potential conflict of interest.
