# Supplementary material for: Functionalized Oxoindolin Hydrazine Carbothioamide Derivatives as Highly Potent Inhibitors of Nucleoside Triphosphate Diphosphohydrolases
Source: Front Pharmacol. 2020 Nov 30;11:585876. doi: 10.3389/fphar.2020.585876 (PMC7734281; doi:10.3389/fphar.2020.585876)
Supplement: Supplementary file 1 [file datasheet1.docx]

Supplementary Material

# Representative ^1^H and ^13^C NMR Spectra:

NMR (including ^1^H and ^13^C NMR) spectra for representative compounds are given below

**8c**

**Supplementary Figure 1.** ^1^H spectra of compound **8c**

**Supplementary Figure 2.** ^13^C spectra of compound **8c**

**8d**

**Supplementary Figure 3.** ^1^H spectra of compound **8d**

**Supplementary Figure 4.** ^13^C spectra of compound **8d**

**8f**

**Supplementary Figure 5.** ^1^H spectra of compound **8f**


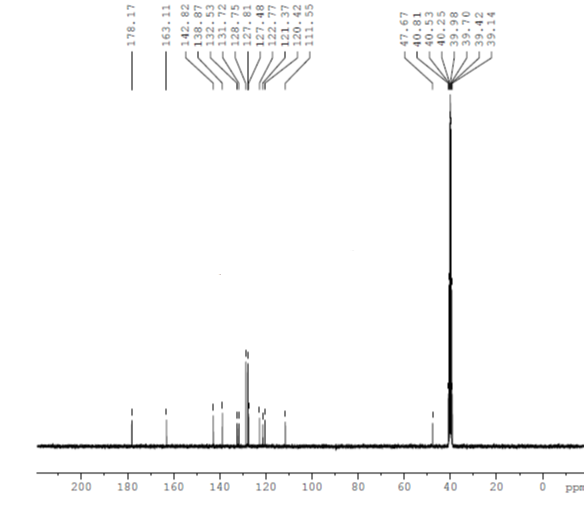


**Supplementary Figure 6.** ^13^C spectra of compound **8f**

**8i**

**Supplementary Figure 7.** ^1^H spectra of compound **8i**

**Supplementary Figure 8.** ^13^C spectra of compound **8i**

**8m**

**Supplementary Figure 9.** ^1^H spectra of compound **8m**


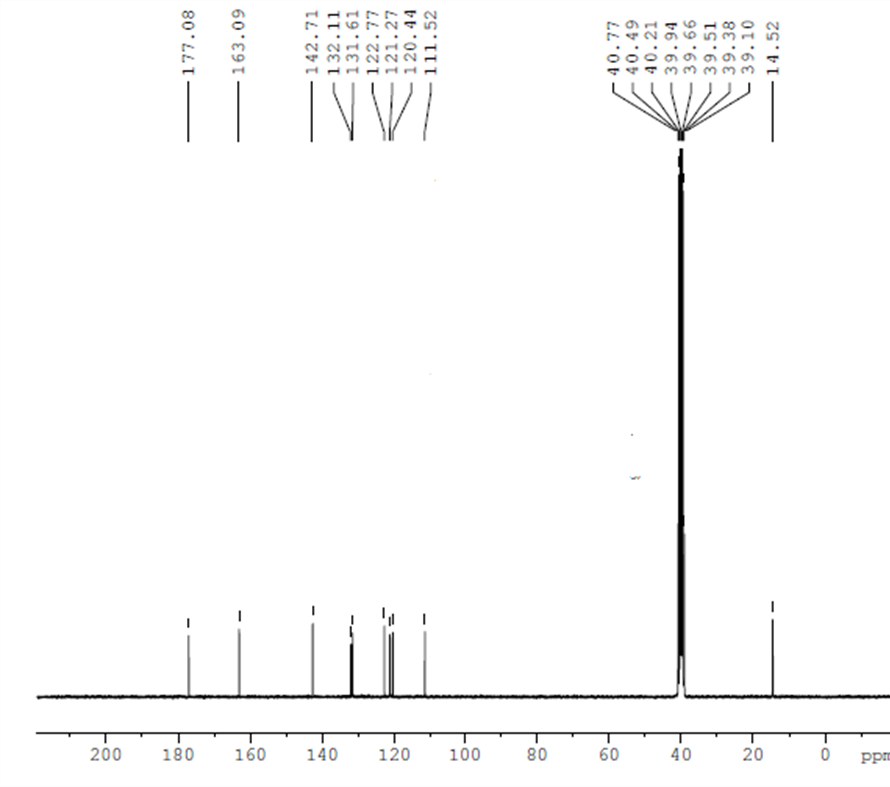


**Supplementary Figure 10.** ^13^C spectra of compound **8m**

HSQC Data of Compound **8i**


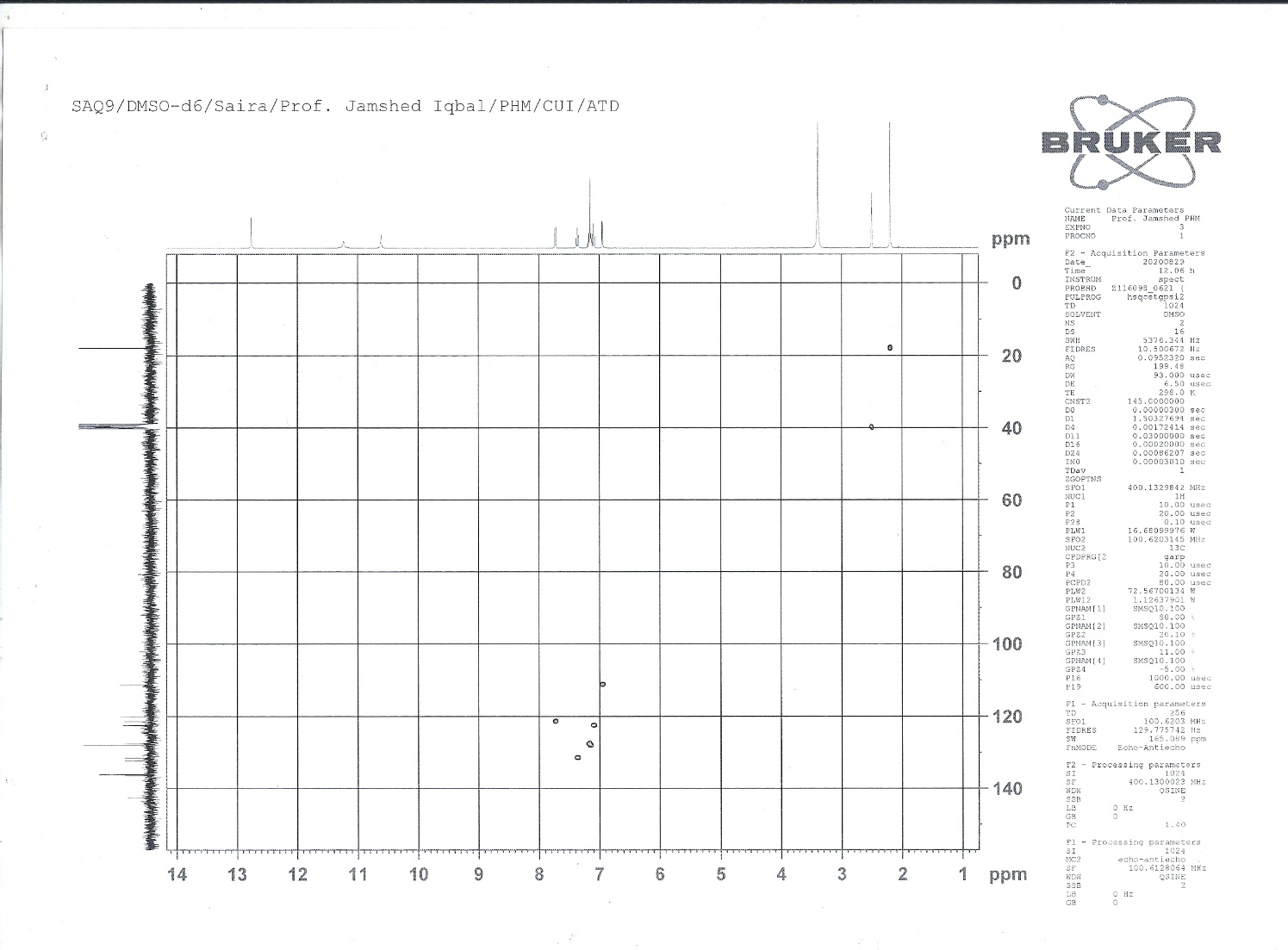


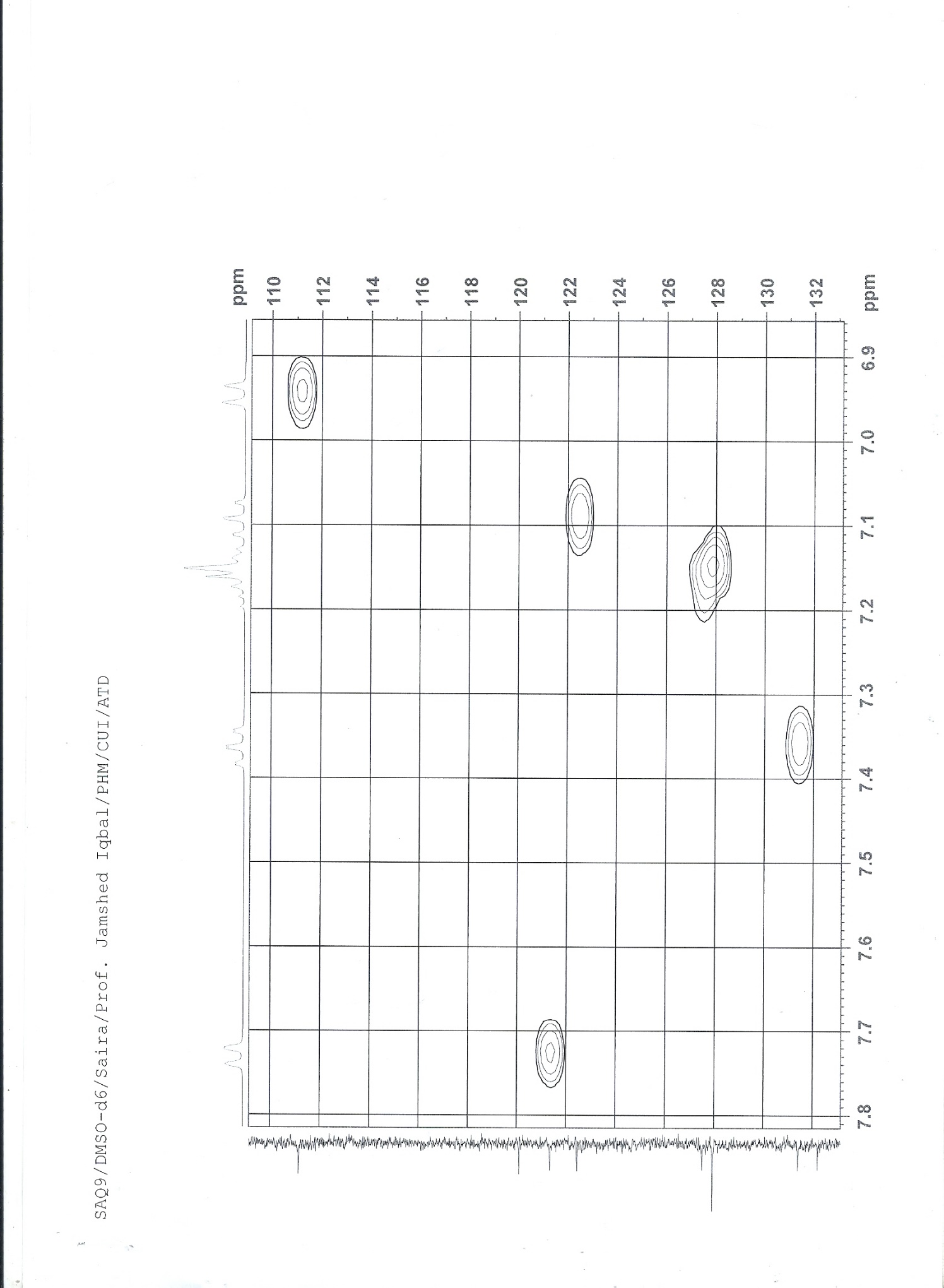

HMBC Data of Compound **8i**
